# Carrier concentration dependence of structural disorder in thermoelectric Sn_1−*x*_Te

**DOI:** 10.1107/S2052252516012707

**Published:** 2016-08-22

**Authors:** Mattia Sist, Ellen Marie Jensen Hedegaard, Sebastian Christensen, Niels Bindzus, Karl Frederik Færch Fischer, Hidetaka Kasai, Kunihisa Sugimoto, Bo Brummerstedt Iversen

**Affiliations:** a Center for Materials Crystallography, Department of Chemistry and iNANO, Aarhus University, Langelandsgade 140, Aarhus C, DK-8000, Denmark; b Faculty of Pure and Applied Sciences, University of Tsukuba, 1-1-1 Tennodai, Tsukuba, 305-8571, Japan; c Japan Synchrotron Radiation Research Institute, I-I-I, Kouto, Sayo-cho, Sayo-gun, Hyogo, 679-5198, Japan

**Keywords:** tin telluride, anharmonicity, maximum entropy method, disorder, synchrotron X-ray diffraction

## Abstract

SnTe is a promising thermoelectric and topological insulator material. Here, the presumably simple rock salt crystal structure of SnTe is studied comprehensively by means of high-resolution synchrotron single-crystal and powder X-ray diffraction from 20 to 800 K. Two samples with different carrier concentrations (sample *A* = high, sample *B* = low) have remarkably different atomic displacement parameters, especially at low temperatures. Both samples contain significant numbers of cation vacancies (1–2%) and ordering of Sn vacancies possibly occurs on warming, as corroborated by the appearance of multiple phases and strain above 400 K. The possible presence of disorder and anharmonicity is investigated in view of the low thermal conductivity of SnTe. Refinement of anharmonic Gram–Charlier parameters reveals marginal anharmonicity for sample *A*, whereas sample *B* exhibits anharmonic effects even at low temperature. For both samples, no indications are found of a low-temperature rhombohedral phase. Maximum entropy method (MEM) calculations are carried out, including nuclear-weighted X-ray MEM calculations (NXMEM). The atomic electron densities are spherical for sample *A*, whereas for sample *B* the Te electron density is elongated along the 〈100〉 direction, with the maximum being displaced from the lattice position at higher temperatures. Overall, the crystal structure of SnTe is found to be defective and sample-dependent, and therefore theoretical calculations of perfect rock salt structures are not expected to predict the properties of real materials.

## Abstract 2

The crystal structure of SnTe is investigated from 20 to 800 K in two samples with different carrier concentrations by single-crystal and powder synchrotron X-ray diffraction, coupled with maximum entropy analysis.

## 1. Introduction   

Group IV chalcogenides such as Pb*X*, Sn*X* and Ge*X* (*X* = S, Se, Te) are currently under intense investigation in materials science since they exhibit a range of extraordinary properties. Several materials (*e.g.* SnTe) have been shown to be topo­logical insulators (Hsieh *et al.*, 2012 ▸), and in the field of thermo­electrics PbTe has been a key material for more than five decades due to its extraordinary high figure of merit, *zT* (Dughaish, 2002 ▸). The high *zT* value is due both to a favorable multi-valley electronic band structure and to an unexpected very low thermal conductivity for a simple rock salt structure (Heremans *et al.*, 2008 ▸). The tin chalcogenides show even better thermoelectric properties and recently SnSe was reported to have a record-breaking *zT* value of 2.6 (Zhao *et al.*, 2014 ▸). Determination of accurate crystal structures is clearly a prerequisite for understanding any of the multitude of attractive properties observed in the group IV chalcogenides (Sist *et al.*, 2016 ▸). These materials are presumed to have simple crystal structures, but this makes it difficult to understand *e.g.* the very low thermal conductivities observed in these materials. Indeed, in the case of PbTe recent work has demonstrated that the crystal structure is much more complex, with substantial disorder and/or strong anharmonicity (Bozin *et al.*, 2010 ▸; Kastbjerg *et al.*, 2013 ▸). Many studies have also carried out theoretical calculations on the group IV chalcogenides in order to understand their properties (Li, Hellman *et al.*, 2014 ▸; Lee *et al.*, 2014 ▸), but such calculations are challenged if in reality the materials have much more complex structures or are highly defective. In the present study, we carry out a comprehensive structural study of a key group IV chalcogenide, SnTe, which has been scrutinized for decades.

Tin telluride is a IV–VI non-stoichiometric narrow-gap semiconductor. Recent experimental findings on size-tunable band gaps in quantum dots (Kovalenko *et al.*, 2007 ▸), on the topological insulator state (Tanaka *et al.*, 2012 ▸) and on its thermoelectric performance (Zhang *et al.*, 2013 ▸; Tan *et al.*, 2014 ▸, 2015 ▸) have fuelled interest in the crystal structure of this material which, at first sight, has a simple rock salt structure, space group 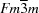. In particular, the origin of its extremely low thermal conductivity has so far been elusive. Recent pair distribution function (PDF) investigations (Knox *et al.*, 2014 ▸) suggest the formation of local dipoles (disorder) between 300 and 400 K. However, inelastic neutron scattering measurements coupled with molecular dynamics calculations (Li, Hellman *et al.*, 2014 ▸) suggest that the thermal motion is anharmonic, without any symmetry breaking on the Sn site. EXAFS experiments, on the other hand, show that SnTe at the local scale is rhombohedrally distorted and that the deviations from cubic symmetry increase for *T* > 100 K (Mitrofanov *et al.*, 2014 ▸). The ongoing debate on the real structure of tin telluride complements fundamental controversies on other chalcogenides such as Pb*X* (*X* = S, Te) and GeTe. In the case of Pb*X*, scattering studies (Bozin *et al.*, 2010 ▸; Kastbjerg *et al.*, 2013 ▸) show an off-centring of Pb in the axial directions, whereas EXAFS (Keiber *et al.*, 2013 ▸) and inelastic neutron scattering investigations (Li, Hellman *et al.*, 2014 ▸) describe the thermal motion of Pb as strongly anharmonic. In GeTe, the displacive nature of the high-temperature phase transition has recently been questioned by EXAFS, PDF and Raman investigations (Fons *et al.*, 2010 ▸; Matsunaga *et al.*, 2011 ▸), which point out that the high-temperature cubic phase is indeed disordered. Again, even for GeTe, the consensus is far from unanimous (Wdowik *et al.*, 2014 ▸; Chatterji *et al.*, 2015 ▸). Concerning SnTe, we also recall the controversial presence of a quasi-second-order phase transition from 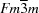 to *R*3*m* in a certain range of carrier concentration. The phase transition was initially suggested by analogy with GeTe and has been the subject of many and often disagreeing studies in the past few decades (Ortalli, 1984 ▸).

In order to unravel the subtle features of the crystal structure of SnTe, we have investigated its structure between 20 and 800 K using single-crystal X-ray diffraction (SCXRD) and powder X-ray diffraction (PXRD) experiments, using both synchrotron radiation and conventional in-house X-ray sources. The recent developments of the maximum entropy method (MEM) are employed on two samples with different carrier concentrations (Christensen *et al.*, 2015 ▸).

## 2. Experimental and methods   

### 2.1. Synthesis of samples *A* and *B*   

In the synthesis of sample *A*, equivalent amounts of semiconductor grade Sn and of Te were pre-reacted in an evacuated quartz ampoule. The synthesized SnTe was repacked into a longer evacuated quartz ampoule and vapour transport synthesis was performed at 1083 K for 10 d.

Sample *B* was synthesized from the direct melting of Sn and Te in a molar ratio of 1.05:1 which, according to Tan *et al.* (2014 ▸), corresponds to the limit of solubility of Sn in SnTe and gives a carrier concentration of around 1.5 × 10^20^ cm^−3^ at room temperature. The homogeneity of this sample was tested by potential Seebeck microprobe measurements (Platzek *et al.*, 2005 ▸).

### 2.2. Sample characterization   

#### 2.2.1. Hall coefficient and resistivity measurements   

Given the small crystal dimensions of sample *A* (∼40 µm equivalent radius), it was not possible to perform Hall coefficient measurements. The carrier concentration at 300 K, *p*_300 K_, of sample *A* was estimated to be 8.0 × 10^20^ cm^−3^ from the cell parameter at room temperature (*a*_0_) through the relation *a*_0_(SnTe) = −1.7 × 10^−23^ Å cm^3^ × *p*_300 K_ + 6.327 Å (Bis & Dixon, 1969 ▸), which was obtained empirically by studying samples with 0.3 × 10^20^ < *p*_300 K_ < 9.5 × 10^20^ cm^−3^.

**Figure 1:**
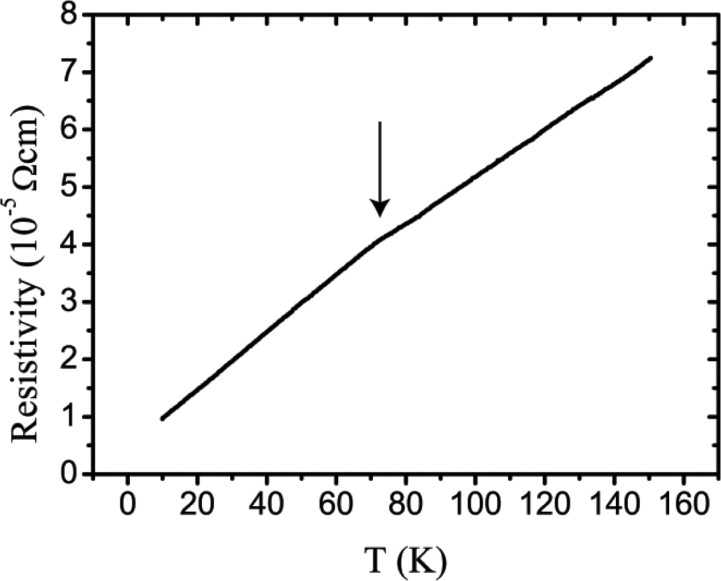
The measured resistivity of sample *B* using the PPMS. The arrow marks a kink in the resistivity. A similar kink was interpreted as evidence of a structural phase transition by Kobayashi *et al.* (1976 ▸).

The large ingot of sample *B* (∼ 6 × 1 × 1 cm) was cut into a small bar on which measurements with a Physical Property Measurement System (PPMS; Quantum Design) were performed. *p*_77 K_ is estimated to be 2.05 × 10^20^ cm^−3^ from PPMS Hall measurements, while *p*_300 K_ is 1.6 × 10^20^ cm^−3^ from Hall measurements using a home-built system (Borup *et al.*, 2012 ▸). The cell parameters confirm that sample *B* has a low carrier concentration, although given the precision of the relation it is not possible to calculate *p*_300 K_ reliably since, for sample *B*, *a*_0_ is 6.327 (2) Å, hence *p*_300 K_ would be zero. There is a general consensus that the potential phase transition temperature, *T*_c_, to the rhombohedral system depends on the carrier concentration. The phase transition can possibly be located by a kink in the resistivity curve *versus* temperature. The extrapolation of the values reported by (Kobayashi *et al.*, 1976 ▸) resulted in a polynomial
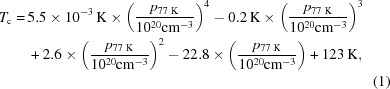
with *T*_c_ ranging from 0 to 123 K for samples with carrier concentrations ranging from 13 × 10^21^ cm^−3^ to 0. Thus, sample *B* should have a *T*_c_ around 86 K. The kink in the resistivity of sample *B* is found at *T* ≃ 78 K (Fig. 1 ▸). All different estimates of the carrier concentration show that sample *B* has a low carrier concentration and that the phase transition should be in the range 75–90 K. A powder sample and a single crystal (equivalent radius ∼25 µm) were obtained from this ingot for the diffraction measurements.

#### 2.2.2. X-ray diffraction   

High-resolution SCXRD data were collected on samples *A* and *B* at SPring8 (beamline BL02B1) with wavelengths of 0.499120 and 0.499718 Å, respectively. A Rigaku Kappa diffractometer equipped with a cylindrical image plate was used to collect the data. Integration of the Bragg reflections, Lorentz-polarization correction, empirical absorption correction (Blessing, 1995 ▸) and scaling were carried out using the *RAPID-AUTO* software (Rigaku Corporation, 2004). The unmerged data were sorted and averaged using the *SORTAV* program (Blessing, 1997 ▸). Values of σ as defined in *SORTAV* were used in the weighting scheme. Crystal structure refinements were carried out using *JANA2006* (Petříček *et al.*, 2014 ▸). The extinction correction resulted in statistically insignificant parameters for sample *B* and nearly insignificant parameters for sample *A*, thus the extinction correction was not applied to the final data to avoid structural bias in the MEM density. The data were corrected for anomalous dispersion (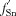 = −1.534, 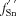 = 0.767, 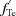 = −1.228, 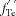 = 0.906). For sample *A*, complete data sets (100%) with maximal sinθ/λ = 1.2 Å^−1^ were collected at 20, 200, 300 and 400 K. Furthermore, small data sets were collected at 50, 75 and 110 K. For sample *B*, complete data sets were collected with maximal sinθ/λ = 1.0 Å^−1^ at 20, 50, 80, 110, 200 and 300 K. Experimental and crystallographic details are given in the supporting information.

PXRD data on sample *B* were collected on an in-house Rigaku Smartlab diffractometer equipped with a Cu source from 300 to 800 K, and at beamline BL44B2 (Kato *et al.*, 2010 ▸; Kato & Tanaka, 2016 ▸) with a nitrogen low-temperature and high-temperature blower at SPring8, Japan, at 120, 200 and 300 K with a wavelength of 0.50036 (7) Å and at 300, 400, 550 and 700 K with a wavelength of 0.50027 (5) Å. Furthermore, PXRD data were collected on sample *B* from 10 to 200 K using a closed-cycle cryostat on beamline BL44B2 with a wavelength of 0.50036 (7) Å. In this case the capillary was enclosed in helium and the sample was placed directly in contact with the copper sample holder, the temperature of which was monitored directly by a thermocouple. Pattern fitting was carried out on the PXRD data to study the cell-parameter and peak-width evolution at low temperature.

### 2.3. Maximum entropy method calculations   

MEM (Sakata & Sato, 1990 ▸) and nuclear-weighted X-ray MEM (NXMEM) calculations (Christensen *et al.*, 2015 ▸) were performed on the single-crystal data collected at 20, 200, 300 and 400 K for sample *A*, and at 20, 50, 80, 110, 200 and 300 K for sample *B*. The observed structure factors on an absolute scale obtained from the harmonic model (see Section 3.3.1) were transformed into pseudo-nuclear structure factors following the NXMEM procedure. The Sakata–Sato MEM formalism (Sakata & Sato, 1990 ▸), as implemented in *BayMEM* (van Smaalen *et al.*, 2003 ▸), was applied to the pseudo-nuclear structure factors to enhance substantially the nuclear density resolution. The unit cell was divided into 256 × 256 × 256 pixels and the calculations were initiated from a uniform prior.

In the MEM formalism the stopping criterion, 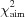, cannot be unequivocally defined (Iversen *et al.*, 1995 ▸; Hofmann *et al.*, 2007 ▸; Bindzus *et al.*, 2015 ▸; van Smaalen & Netzel, 2009 ▸). Furthermore, data collected at different temperatures exhibit different significances (*F*/σ) as a function of sinθ/λ. This implies that a different resolution-dependent fitting occurs in the final MEM density. In the NXMEM algorithm, the error inherent in the deconvolution procedure is unknown, which makes the weighting scheme intrinsically less reliable. In the present work, only flat prior densities were used in order to minimize structural bias. Different stopping criteria in the MEM calculations were tested, 0.2 ≤ 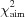 ≤ 20 (Bindzus & Iversen, 2012 ▸). For both samples we note that the residuals in the Fourier difference map and the values of the electron densities exhibit an asymptotic behaviour on lowering the final 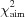 value. Consequently, MEM densities with 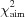 = 0.2 are reported here. In the NXMEM computations, the Fourier difference values are much higher and present a greater variability with temperature. Low values of the constraint are difficult to achieve and 1 ≤ 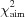 ≤ 10 have been tested. 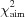 at 20 K has been set to 1 and 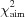 at the other temperatures have been chosen so that the Fourier residuals in the final NXMEM density remain in the same range as at 20 K. Although this choice is somewhat arbitrary and it affects the final density quantitatively, it does not alter qualitative conclusions such as the trend of the electron-density maxima and the aspherical features of the MEM and NXMEM densities, which are observed regardless of the tested 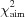 value.

## 3. Results and discussion   

### 3.1. Microstrain, mosaicity and diffuse scattering   

**Figure 2:**
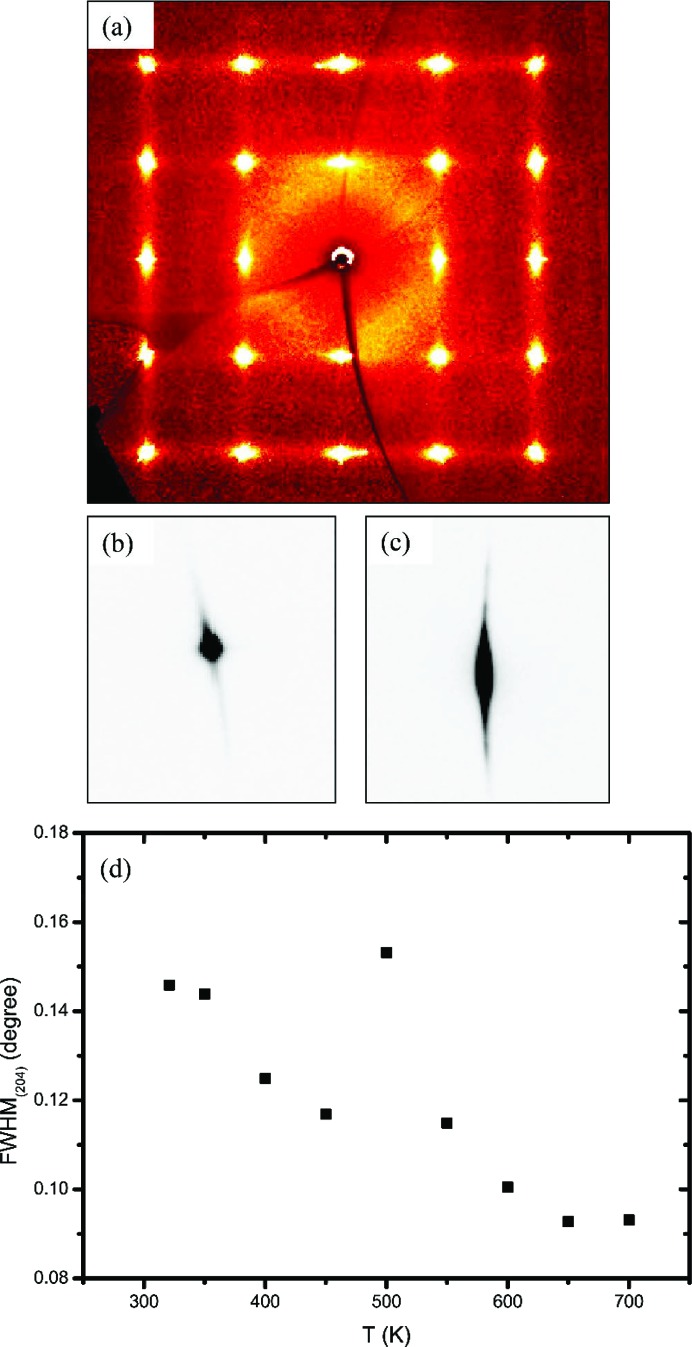
(*a*) X-ray diffuse scattering from sample *A* at 300 K in the (*hk*0) plane; the same diffuse scattering pattern is observed for sample *B*. (*b*) The (422) reflection at 20 K for sample *A*, collected with the image plate on beamline BL02B1. (*c*) The (422) reflection at 20 K for sample *B* on the same intensity scale as in part (*b*), collected with the image plate on BL02B1. (*d*) The FWHM of the (204) reflection plotted as a function of temperature from powder X-ray diffraction (PXRD) (sample *B*) derived from conventional data measured on a Rigaku SmartLab diffractometer. At 500 K an increase in the FWHM reflects the formation of multiple peaks and asymmetries, as discussed in Section 3.2.

As a first approach, a direct inspection of the diffraction frames provides valuable information. Clear signs of high mosaicity are present in the single-crystal diffraction patterns of both sample *A* and sample *B*, with the Bragg peaks being both broad and long (Fig. 2 ▸). It can, however, be noted that sample *B* shows a much higher degree of mosaicity, probably due to the different sample-preparation procedure. This feature was observed in the diffraction patterns of all the tested sample *B* crystals (∼60 crystals). In addition, the powder diffraction pattern of sample *B* shows clear signs of peak broadening. An analysis carried out with *WinPLOTR* (Roisnel & Rodriguez-Carvajal, 2001 ▸) using LaB_6_ as a standard material indicates that the larger contribution to peak broadening is due to microstrain effects, which account for local differences in the cell parameters. Local differences in the cell parameters are also corroborated by the applicability of Vegard’s law with Sn content (Bis & Dixon, 1969 ▸; Mikkelsen & Boyce, 1982 ▸). This feature is likely related to the non-stoichiometry in SnTe and it is common to IV–VI non-stoichiometric compounds such as SnSe, PbS, PbSe and PbTe (Sist *et al.*, 2016 ▸; Christensen *et al.*, 2016 ▸).

For both samples, the single-crystal diffraction patterns present diffuse scattering consisting of planes connecting the reciprocal lattice points through the 〈100〉 directions, which is indicative of correlated disorder (static or dynamic). The diffuse scattering is clearly visible for *T* > 100 K, and even at 20 K it is faintly visible. For both samples, together with the increase in diffuse scattering, there is a dramatic loss in intensity at high resolution for *T* > 100 K which is modelled with increased atomic displacement parameters (ADPs) in the structural refinements.

### 3.2. Cell parameters   

**Figure 3:**
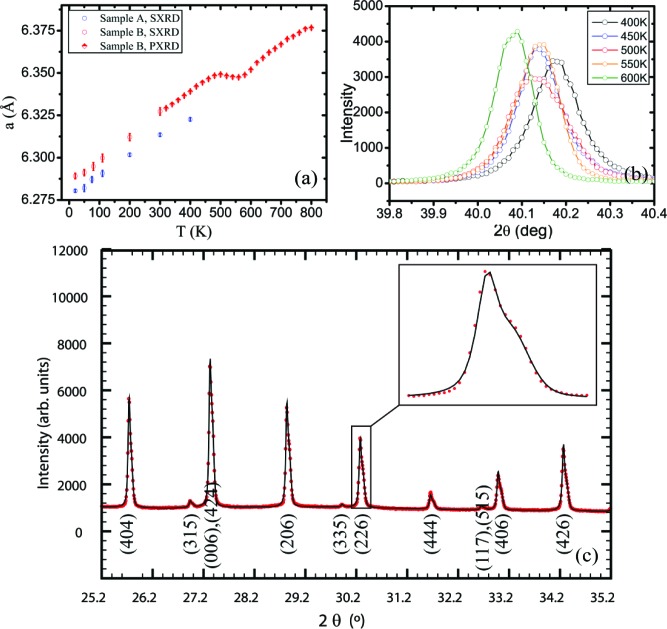
(*a*) The cell parameters of samples *A* and *B* from synchrotron single-crystal X-ray diffraction (SXRD) and conventional powder X-ray diffraction (PXRD). (*b*) The (204) reflection plotted as a function of temperature collected with a conventional X-ray source (sample *B*). (*c*) A high-resolution synchrotron PXRD pattern showing asymmetries at 550 K (sample *B*).

Tin telluride is non-stoichiometric, and the ratio of Sn:Te is always less than one. The effect of each Sn vacancy is the creation of two electron holes, rendering tin telluride a *p*-type semi-metal (Salje *et al.*, 2010 ▸), *i.e.* a zero-gap semiconductor, due to the small overlap between the bottom of the conduction band and the top of the valence band. The carrier concentration ranges from 10^19^ to 10^21^ cm^−3^. Crystals with a low carrier concentration (fewer Sn vacancies) have relatively larger cell parameters (Bis & Dixon, 1969 ▸). The cell parameters determined for the two different samples reflect the preparation method employed. The vapour transport synthesis is more prone to giving samples with a low tin content, due to the higher vapour pressure of tellurium. The opposite happens when the sample is synthesized by directly melting Sn in excess and Te. As shown in Fig. 3 ▸, the cell expansion is linear in the range 20–400 K for both samples, the slope being slightly different in the two cases. For sample *B*, the cell volume does not vary appreciably in the range 450–550 K. The clear broadening of the Bragg peaks at 500 K indicates a conspicuous increase in microstrain. The appearance of shoulders and asymmetries for *T* ≥ 500 K can be ascribed to the formation of multiple phases with different contents of tin and hence with different carrier concentrations. Above 700 K a further broadening is detected and the scattering power decreases due to the formation of SnO_2_. The cell expansion curve is not reversible in the sense that, upon cooling, the cell parameters are systematically lower than on warming. The broadness of the peaks and the presence of multiple phases with slightly different unit-cell volumes persist even at room temperature. However, the trend shown in Fig. 3 ▸ is not entirely general since, on increasing the temperature ramping rate or the time of acquisition at each temperature, the formation temperature of multiple phases increases and the cell thermal expansion changes accordingly.

### 3.3. Atomic displacement parameters and Sn occupancy   

Three different structural models were tested: (i) both Sn and Te treated with a harmonic thermal motion (harmonic model); (ii) refinement of fourth-order Gram–Charlier co­efficients *D*_1111_ and *D*_1122_ (Kuhs, 1992 ▸) for one atom while keeping the other harmonic; and (iii) refinement of the Gram–Charlier coefficients for both Sn and Te. In all models, the occupancy of Sn was refined separately for each temperature.

#### 3.3.1. Harmonic model   

If a harmonic thermal motion is assumed, the cubic symmetry constrains the ADPs of both Sn and Te atoms to be isotropic.

**Figure 4:**
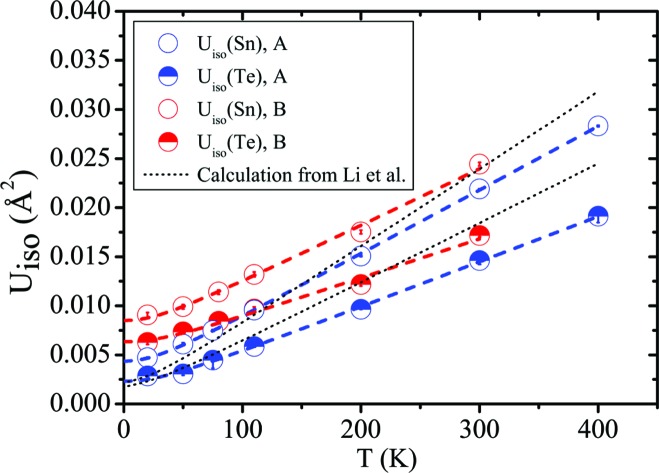
The ADPs of Sn and Te for samples *A* and *B* from synchrotron SXRD in comparison with the theoretical values from Li, Ma *et al.* (2014 ▸). The coloured dashed lines represent fits to a Debye model.

Fig. 4 ▸ shows the thermal behaviour of the Sn and Te ADPs in the two samples. Correlation coefficients between *U*_iso_(Sn) and *U*_iso_(Te) range from 0.96 to 0.77 at 20 and 400 K, respectively, in sample *A*, and from 0.94 to 0.87 at 20 and 300 K, respectively, in sample *B*. Sn has a higher isotropic ADP than Te in both samples, which implies that the nuclear probability density function of Sn is more diffuse. The absolute difference increases with temperature. The trend of *U*_iso_(Sn) and *U*_iso_(Te) with temperature in sample *B* matches the experimental findings of a recent study (Li, Ma *et al.*, 2014 ▸) and is in disagreement with the theoretical values provided in the same study. The ADPs of both atoms in sample *A* are fairly close to those of sample *B* at room temperature. However, their decrease with decreasing temperature is much more marked, to the point that, at 20 K, *U*_iso_(Sn) and *U*_iso_(Te) are half the values of sample *B*. It is worth stressing that the difference is already clear at 110 K, which is above the reported phase transition. The Debye expression (Willis & Pryor, 1975 ▸) can be used to model the lattice dynamics of SnTe:
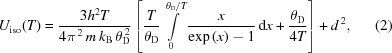
where *U*_iso_(*T*) is the weighted isotropic ADP, θ_D_ is the Debye temperature, *m* is the mass of Sn or Te and *d* ^2^ is a disorder parameter. *d* ^2^ is 0.0021 (1) and 0.0005 (2) Å^2^ for Sn and Te, respectively, in sample *A*, and 0.0064 (3) and 0.0046 (3) Å^2^ for Sn and Te, respectively, in sample *B*. It should be stressed that *d* should presumably be temperature-independent, which is in contrast with the findings of Knox *et al.* (2014 ▸). It is, however, instructive to notice that in both samples *d* is significantly different from zero. Li, Ma *et al.* (2014 ▸) suggested that this might be due to an anharmonic potential-energy curve with a shallow double well, whereas at high temperatures a harmonic ADP is expected since most of the thermal modes behave harmonically.

#### 3.3.2. Anharmonic model   

**Figure 5:**
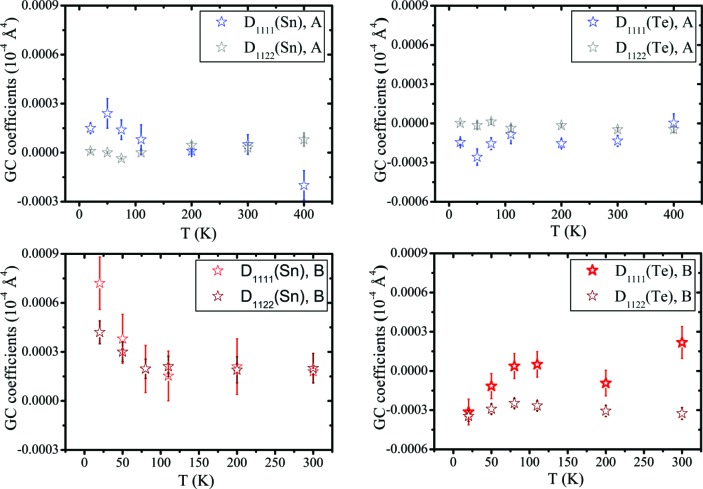
Gram–Charlier parameters of samples *A* and *B* when only one atom is refined anharmonically while the other is kept harmonic.

Anharmonic features can be probed either by refinement of Gram–Charlier (GC) co­efficients (Kuhs, 1992 ▸) or with descriptions based on physical models (Bentien *et al.*, 2002 ▸). Here, we use the GC expansion of the harmonic temperature factor (Fig. 5 ▸). Since both the Sn and Te sites have 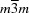 point symmetry, the GC coefficients are constrained to be *D*_1111_ = *D*_2222_ = *D*_3333_ and *D*_1122_ = *D*_1133_ = *D*_2233_. For sample *A*, the anharmonicity is marginally significant for both Sn and Te. Correspondingly, almost-spherical nuclear probability density functions are expected. When the GC coefficients of Sn and Te are refined simultaneously, a high correlation (>90%) between the two parameters occurs.

Sample *B* presents a different thermal behaviour. Again, high correlations prevent a robust description of the thermal motion when GC coefficients of both Sn and Te are refined simultaneously. However, when GC coefficients of only one atom are refined while keeping the other harmonic, then the Sn atom shows a considerable increase in anharmonicity at *T* < 80 K, particularly for *D*_1111_. The fact that the thermal motion becomes anharmonic at lower temperatures is rather unusual. This anomaly may possibly anticipate the phase transition which, however, would then occur at a much lower temperature than the observed kink in the resistivity. It is worth stressing that, at 20 K, the nuclear probability density function of Sn (see supporting information) displays some weak features along the 〈100〉 direction, which does not support any rhombohedral distortion down to this temperature. If only the Te atom is refined anharmonically, *D*_1111_ exhibits large standard deviations, whereas *D*_1122_ is more significant and always negative. However, it should be noted that the nuclear probability density function becomes unphysically negative at the Te position. As in the case of Sn, the probability density function of Te has features along the 〈100〉 direction. In general, for the low carrier concentration sample (*B*), different models agree that the probability density function of Sn or Te, or possibly both, are elongated along the 〈100〉 direction, although the extremely low intensity of the (*hkl*) reflections with *h*, *k*, *l* all odd (proportional to the difference in scattering between the cation and the anion), and the high correlations, prevent a robust quantification of the GC coefficients based on the present single-crystal X-ray diffraction data.

#### 3.3.3. Occupancy of Sn   

**Figure 6:**
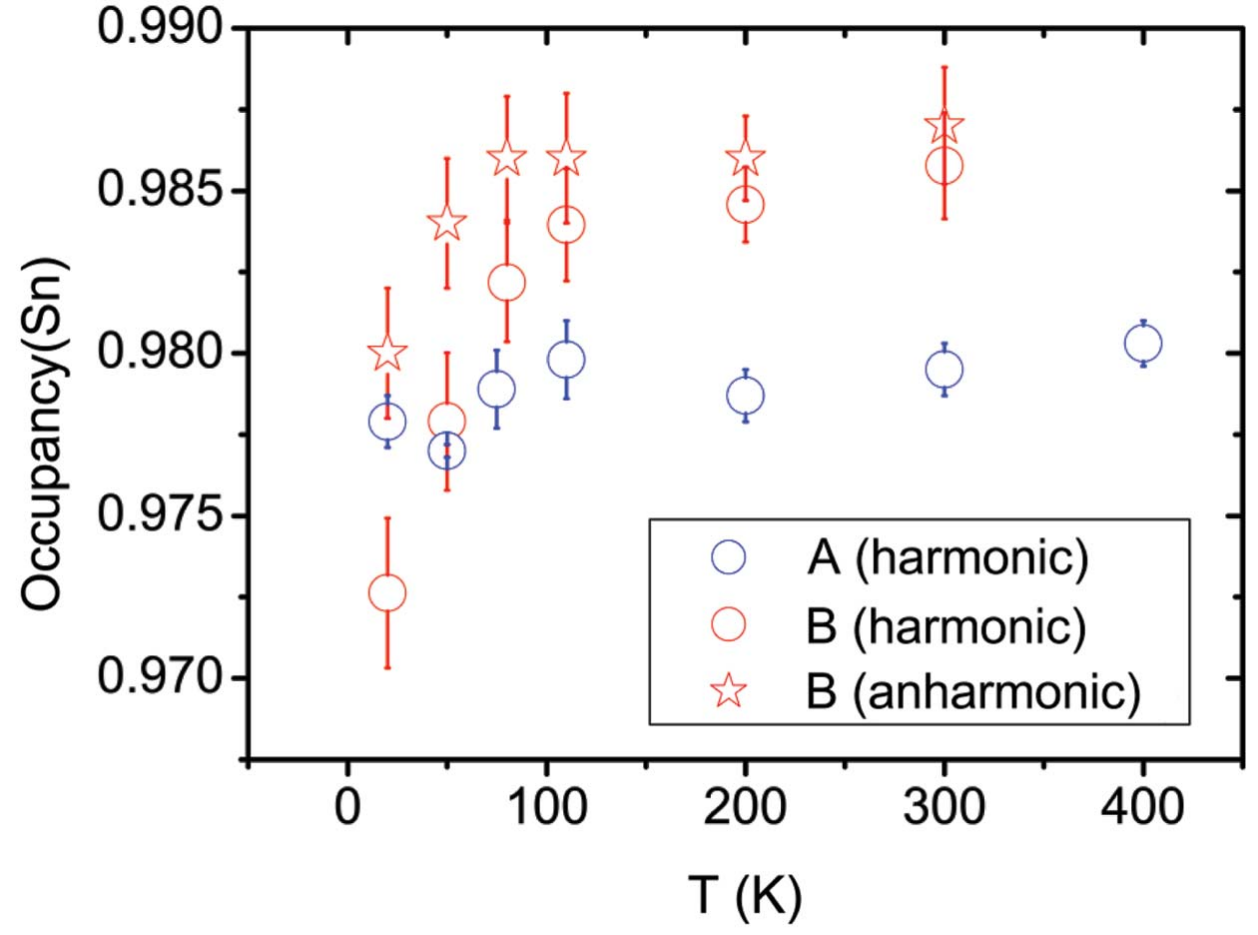
The Sn occupancy in samples *A* and *B* from synchrotron SCXRD when only Sn is refined anharmonically.

The stoichiometry of Sn is an important parameter in dictating the properties (Tan *et al.*, 2014 ▸). The *p*-type behaviour of SnTe is caused by Sn vacancies (Brebrick, 1963 ▸), and the question of whether vacancy ordering occurs has been discussed previously (Nashchekina *et al.*, 1999 ▸, 2008 ▸). Fig. 6 ▸ shows the occupancy of Sn as a function of temperature for the two samples. While for sample *A* the occupancy is nearly constant with temperature (∼2% vacancies), for sample *B* there is a a jump in the range 20–110 K in the harmonic model. For *T* > 110 K, the occupancy is again constant (∼1.5% vacancies). Correlation coefficients between *U*_iso_(Sn), *U*_iso_(Te) and the site occupation factor of tin, s.o.f.(Sn), are lower than 0.6 at all temperatures. When GC coefficients for Sn are implemented, the jump becomes smaller although still significant. The observed trend could be due to an inadequacy of the structural model. Rearrangements of defects at such low temperatures, as well as decreasing numbers of vacancies with increasing temperature, are rare. In addition, we cannot exclude the possibility that the contribution of the diffuse scattering intensities in the integration and their change with temperature might have an effect on the refined s.o.f.(Sn).

Nevertheless, it has been reported (Brebrick, 1963 ▸) that deviations from stoichiometry are likely due to Sn vacancies, but that the presence of further Te interstitials is necessary to explain the discrepancy between the crystallographic density calculated from the lattice parameter and the density obtained by direct experimental measurement. In the same work, we notice that this difference increases going towards higher carrier concentrations, which means that a larger amount of additional tellurium must be present in the lattice. No Te interstitials are seen in the Fourier difference maps. A possible explanation for this apparent contradiction is that the higher carrier concentration in sample *A* is due to Sn vacancies and additional anti-site tellurium defects, with the ratio of vacancies and anti-site tellurium then being different in sample *B*. The increase in Sn occupancy for sample *B* at low temperature may entail some kind of rearrangement of vacancies and anti-site tellurium occurring, in correspondence with the range of temperatures at which a phase transition has been reported. This also coincides with the kink in the resistivity data and might infer a change in the local structure, but it is not related to a transition in the average crystal structure (Galoisy, 1996 ▸; Fons *et al.*, 2010 ▸) as seen by diffraction, which remains cubic.

### 3.4. Maximum entropy method   

**Figure 7:**
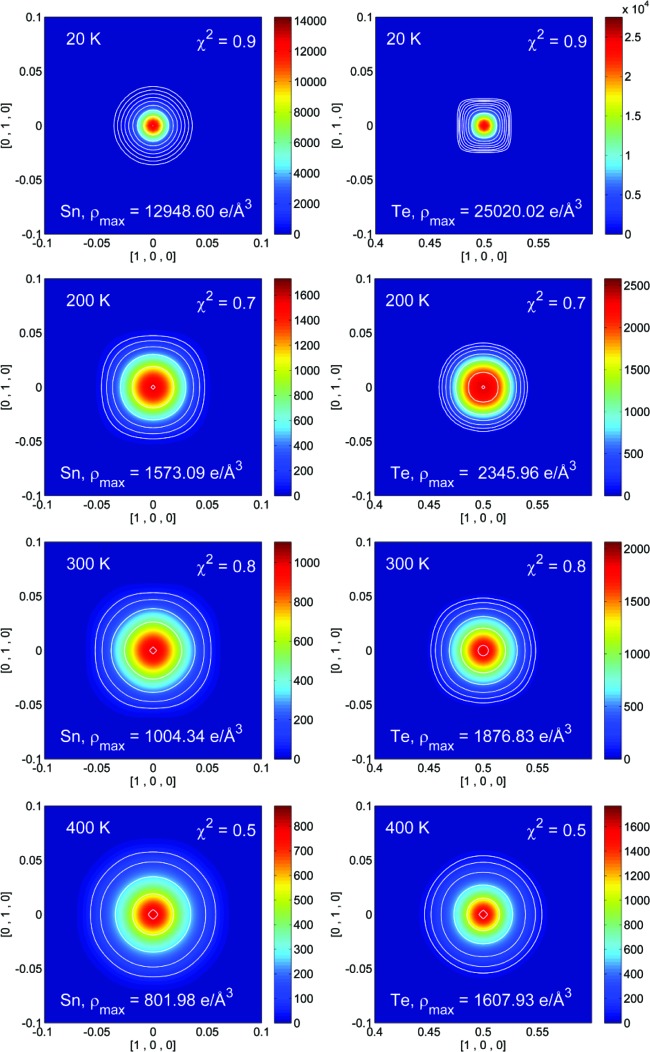
NXMEM electron-density maps for Sn (left) and Te (right) in the (001) plane from 20 to 400 K for sample *A*. Contour lines have been set at 64, 128, 256, 512, 1024, 2048, 4096 and 8192 e Å^−3^. An additional contour line has been added as a guide to locate the maximum corresponding to the nuclear position.

Significant correlations are present when GC coefficients are refined for both Sn and Te. Conversely, the MEM offers a non-parametrized description of the electron or nuclear density (Sakata & Sato, 1990 ▸; Collins, 1982 ▸), and MEM density maps are provided in the supporting information. Recently, the NXMEM procedure has been shown to enhance the nuclear resolution substantially, and thus to enhance the ability to quantify subtle disorder features (Christensen *et al.*, 2015 ▸). Therefore, our study focuses on the NXMEM results (Figs. 7 ▸ and 8 ▸). In both samples *A* and *B* the electron density on the Sn site is a maximum at 20 K and decreases with increasing temperature. Compared with Te, the electron density of the Sn atom is lower and more diffuse. This is in close agreement with the higher ADPs refined for Sn in the least-squares modelling.

For sample *A*, no appreciable aspherical features are observed in the range 20–400 K in either the MEM or the NXMEM maps. Therefore, no conclusions can be drawn on the presence of strong anharmonicity in the 〈100〉 direction and/or static disorder. The very diffuse and spherical electron density indicates that the displacement of the Sn atom is non-directional, whether of a static or dynamic nature. For sample *B*, even at 20 K, the Te electron density is elongated along 〈100〉, whereas this is not the case for the Sn atom. The features on the Te atom increase with temperature. Eventually, at 300 K, the maxima in the NXMEM map are not on the 4*b* position, although a significant amount of electron density is retained at (0.5, 0.5, 0.5). This may indicate that the Te atom moves in a double-well potential or that at 300 K the structure is disordered, with some Te atoms sitting on the 4*b* position and some displaced in the 〈100〉 direction. Given the high-temperature trend of the cell parameter (Fig. 3 ▸), the second hypothesis seems to be more likely. At *T* > 400 K the crystal structure collapses, with the formation of phases with different compositions. It can therefore be argued that, already at 300 K, domains with different carrier concentrations have formed. It has been suggested that, in rock salt structures without vacancies on either site, excess anharmonic motion is not expected along the axial directions since these are the hard potential directions (Kastbjerg *et al.*, 2013 ▸). On the other hand, it seems reasonable that non-spherical features are observed on Te in response to appreciable Sn vacancies, since a lack of Sn atoms will create a softer potential.

### 3.5. On the existence of the *R*3*m* phase   

It is currently accepted that samples with low carrier concentration, such as sample *B*, should become rhombo­hedral at some finite temperature (Shen *et al.*, 2014 ▸), and in the present case this is expected at around 80 K. In other words, samples with a high carrier concentration, and thus a high number of defects, are pinned to the cubic structure, whereas more perfect crystals should convert to the rhombo­hedral structure at low temperature. The existence of a phase transition in SnTe was initially proposed by Stiles & Esaki (1966 ▸) in an attempt to explain the Shubnikov–de Haas effect (Burke *et al.*, 1965 ▸). GeTe transforms from *R*3*m* to 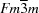 at 660 ≤ *T* ≤ 730 K, depending on the Ge content (Chattopadhyay *et al.*, 1987 ▸). In addition, GeTe–SnTe forms a solid solution, with a phase transition occurring at a lower temperature the higher the content of Sn (Bierly *et al.*, 1963 ▸). A rhombohedral phase in SnTe might thus be expected. However, as early as the 1960s a debate arose on the existence of the phase transition, on the transition temperature and on the origin of the phase transition. For example, the temperature dependence of strain in the 〈110〉 direction does not support any phase transformation down to 1.3 K on a sample with *p*_?_ = 2 × 10^20^ cm^−3^ (here, *p*_?_ is used when we could not find at which temperature the Hall coefficient measurement was carried out) (Stiles & Esaki, 1966 ▸). No sign of any discontinuity has been reported in the elastic constants as a function of temperature, even for samples with extremely low carrier concentrations (Salje *et al.*, 2010 ▸). The first experimental studies of the phonon dispersion relations in SnTe revealed a softening of the transverse optical phonon at the Γ point, but with ω_TO_ remaining finite down to 10 K (Pawley *et al.*, 1966 ▸). For this reason, SnTe was defined as a ‘near ferroelectric’. In a later Raman investigation (Brillson *et al.*, 1974 ▸), the diagonalized polarized longitudinal optical phonon (ω_LO_ = 130.3 cm^−1^) scattering from the (111) SnTe surface was measured at 120 K for samples with *p*_?_ = 1.5 × 10^20^ cm^−3^. It was noted that the Raman line width remains constant and broad, whereas this is expected only near the transition temperature. Furthermore, in the same experiment, the LO phonon scattering exhibits a maximum at 68 K and almost disappears for lower temperatures. The presence of multiple domains or the possible presence of more than one phase transition were hypothesized. A successive Raman study on a sample with *p*_?_ = 1.1 × 10^20^ cm^−3^ showed that several peaks, including one at 300 cm^−1^, persist up to room temperature (Sugai *et al.*, 1977 ▸). It was hypothesized that these Raman peaks are due to lattice vibrations localized around Sn vacancies. In a rock salt structure all the optical vibration modes are Raman inactive. It seems further Raman investigations are called for.

**Figure 9:**
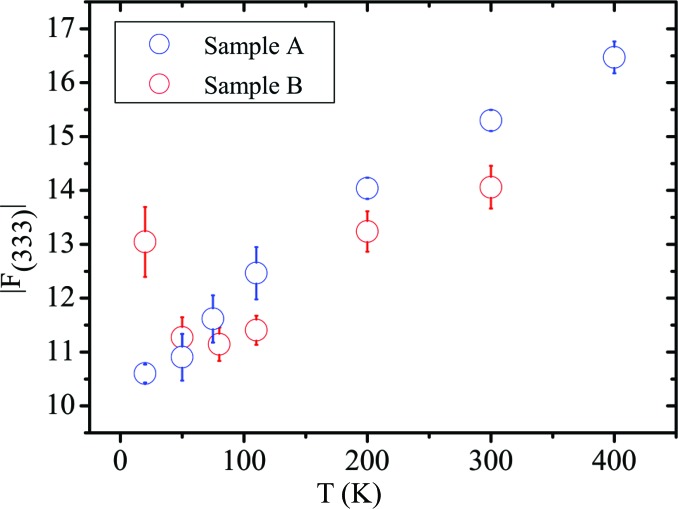
The temperature dependence of *F*_333_ on an absolute scale for samples *A* and *B* from single-crystal diffraction data.

**Figure 10:**
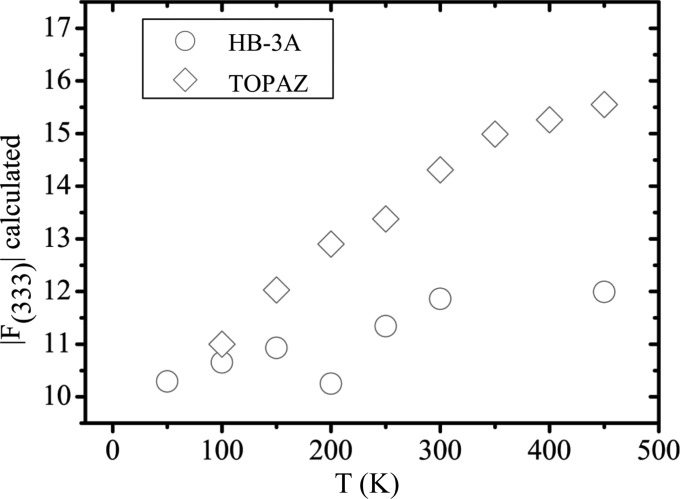
The temperature dependence of *F*_333_ on an absolute scale calculated from *U*_iso_(Sn) and *U*_iso_(Te) reported by Li, Ma *et al.* (2014 ▸)

The diffraction studies supporting the phase transition are from the 1970s. In 1975, a neutron study (Iizumi *et al.*, 1975 ▸) measured the (333) reflection on a sample with *p*_77K_ = 0.88 × 10^20^ cm^−3^. The (333) reflection appeared to fall in intensity when increasing the temperature above 98 K. Since in a rock salt structure, all the structure factors with *hkl* all odd are proportional to the difference in the scattering lengths or form factors of the cation and the anion, it was speculated that the decreasing intensity of the (333) reflection results from centring of the Sn and Te atoms from a rhombohedral to a cubic lattice. Given that (i) the intensity was not reported on an absolute scale, (ii) the effect of the thermal motion was deliberately neglected and (iii) the setting of the diffractometer was not changed to follow the peak position between 20 and 100 K as mentioned by the authors, we feel that their evidence is not entirely persuasive. As a counterproof, we plot the (333) reflection as a function of temperature from the present data in Fig. 9 ▸. The (333) reflection does not approach zero above 100 K but depends on the different increment of *U*_iso_(Sn) and *U*_iso_(Te) with temperature and on the occupancy of Sn. The increase of the *hkl* all odd X-ray structure factors with temperature is confirmed if the values of *U*_iso_(Sn) and *U*_iso_(Te) obtained from neutron measurements at HB-3A and TOPAZ reported by Li, Ma *et al.* (2014 ▸) on a sample with *T*_c_ = 42 K are used to calculate the (333) structure factor (Fig. 10 ▸).

Recent powder diffraction studies have been unable to detect the phase transition, the reason being insufficient peak resolution (Salje *et al.*, 2010 ▸; Li, Ma *et al.*, 2014 ▸). If the rhombohedral angle is too close to 60°, and the Bragg peaks are broad as in the present compound, the superposition of non-equivalent reflections in a powder diffraction pattern becomes unavoidable. In the present study, both samples are cubic at all the temperatures considered, the rhombohedral angle being 60° [α_rh_ = 60.007 (19)°] even at 20 K, as determined from SCXRD experiments.

In a rock salt structure the Laue class 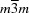 imposes a multiplicity of 48 for a general reflection *hkl*, whereas the multiplicity in the Laue class 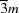 is 12. This implies that, even if the *d* spacing of two non-equivalent reflections in *R*3*m* is virtually the same, thus mimicking a cubic cell, the intensity of certain reflections that are equivalent in 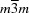 will not be equal in *R*3*m*. Considering a maximum resolution of 0.5 Å, the merging *R* factor 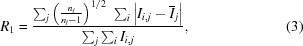
does not vary significantly in all the data sets collected at different temperatures (see supporting information).

**Figure 11:**
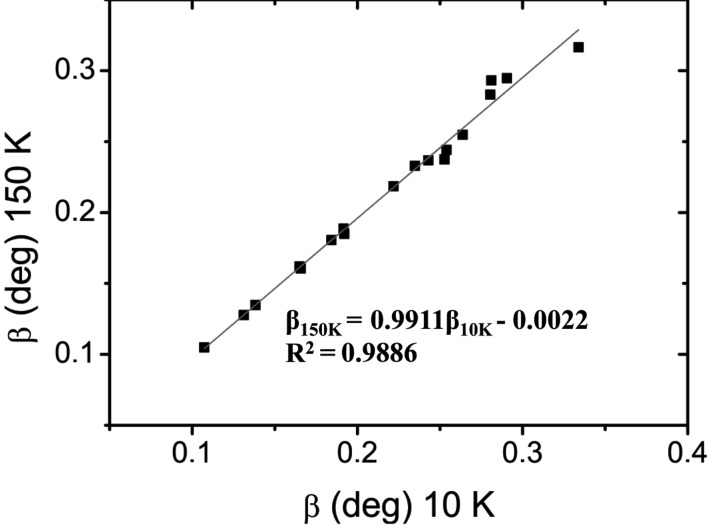
The integral breadth at 150 K *versus* that at 10 K for reflections in the range 12.9 < 2θ < 46.8° [λ = 0.50036 (7) Å] with no peak overlap, and that are supposed to split in a transition 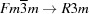.

Finally, as shown by the MEM and NXMEM maps, the electron density on the Sn atom sharpens the lower the temperature and no features appear along the 〈111〉 direction. Again, this tends to support the hypothesis that the structure is cubic at 20 K. Pseudomerohedral twinning of the rhombo­hedral lattice on a cubic lattice leads to perfect Laue symmetry 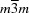. Therefore, to check further for the presence of a phase transition we have measured powder X-ray diffraction on the same powder specimen of sample *B* from 200 K to 10 K and back to 200 K, employing a closed-cycle cryostat. No peak splitting occurs down to 10 K (see supporting information) and the integral breadths at 150 and 10 K appear to be almost unchanged (Fig. 11 ▸). The integral breadths β obtained by fitting single peaks at different temperatures, not corrected for instrumental broadening, show a slight increase below 100 K (see supporting information). However, we notice that such broadening appears mainly at low angles and it occurs not only for those reflections that split if the cubic cell has distorted, but also for the (00*l*) reflections which should remain unaltered if the transition 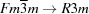 has occurred, *e.g.* (002) and (004). This means that the broadening is more generally ascribable to strain, which could be caused by changes in the defect distribution or by a thermal gradient between the side of the capillary in contact with the copper sample holder and the one that is not.

The appearance of local or submicron rhombohedral distortions on warming to *T* > 100 K (Mitrofanov *et al.*, 2014 ▸) for the Sn sublattice are not supported or disproved by our data, due to the averaging effects intrinsic to diffraction. However, it can be argued that the coherence length of the distortion must be small enough so that no features along 〈111〉 are seen with diffraction techniques. The presence of a phase transition down to 20 K is therefore not confirmed by the present data, even if a small kink in the resistivity is observed.

## 4. Conclusions   

The structure of SnTe has been studied from 20 to 800 K by means of powder and single-crystal synchrotron X-ray diffraction. We have investigated two samples with high (sample *A*) and low (Sample *B*) carrier concentrations. Sample *B* exhibits the well known kink in resistivity at *T* = 78 K. The results of the present study can be summarized as follows.

(i) Overall, both samples exhibit high mosaicity and strain. Diffuse scattering is barely detectable at 20 K, but grows significantly between 50 and 80 K.

(ii) The different cell parameters for samples *A* and *B* reflect the different carrier concentrations. For *T* > 400 K, the clear appearance of multiple phases with different cell parameters accounts for the formation of regions enriched in Sn and others in Sn vacancies. The temperature of this transition depends on the heating rate and the transition is irreversible.

(iii) Over the temperature range 20–400 K, *U*_iso_(Sn) is always considerably larger than *U*_iso_(Te). This is in agreement with recent experimental studies by Li, Ma *et al.* (2014 ▸) but in contrast with Knox *et al.* (2014 ▸). *U*_iso_(Sn) and *U*_iso_(Te) increase linearly with temperature, although the slope is higher for *U*_iso_(Sn). Sample *B* has much larger ADPs than sample *A* at low temperature, and this may reflect static disorder. In all structural models, the occupancy of the Sn atom increases between 20 and 80 K in sample *B*. This subtle behaviour may be related to the presence of some additional Te in the lattice and to defect rearrangements. All these observations are consistent with the real structure of sample *B* being different from that of sample *A*, *i.e.* with more, different and temperature-dependent defects. The nature of such defects and their influence on the ADPs need further investigation.

(iv) The implementation of anharmonic Gram–Charlier coefficients in refinement of the SCXRD data (20–300 K) results in large parameter correlations. If only the Sn atom is refined anharmonically, the GC coefficients are not significant in sample *A*, whereas they are significant for sample *B* at low temperature. For sample *B*, the GC coefficients of Te are significant at all temperatures and particularly at 300 K.

(v) Overall, the MEM and NXMEM maps show a diffuse electron density on the Sn site, while the density is higher on the Te atom. In sample *A* no deviations from sphericity are observed. In sample *B*, on warming, strong features appear in the 〈100〉 direction for the Te atoms. At 300 K, the maximum of the electron density is not on the Te site but is displaced by 0.12 Å. This presumably reflects the incipient formation of multiple phases observed at high temperatures. The disorder observed on the Te site may be related to the presence of Sn vacancies, which cause the Te atom to displace from the high-symmetry position.

(vi) Despite the kink in resistivity observed for sample *B* at *T* = 78 K, the average structure as probed by diffraction (Galoisy, 1996 ▸) remains cubic down to 20 K, within the precision of the present experiment. The increase in intensity of the (*hkl*) reflections all odd with temperature cannot be used as a criterion to judge the existence of the phase transition 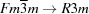, since it is related to the different increases in the ADPs of Sn and Te with temperature.

(vii) It has been reported that the carrier concentration is instrumental in dictating the thermoelectric properties of SnTe (Tan *et al.*, 2014 ▸). The present work shows that the structure and stability of SnTe are highly dependent on the carrier concentration, which has to be considered in further discussions on anharmonicity/disorder within this compound. Furthermore, the intrinsic non-stoichiometry of SnTe should be taken into account in theoretical calculations and the presence of vacancies might contribute significantly to lowering the bulk thermal conductivity.

(viii) Since the physical properties of SnTe are highly sample-dependent, it appears questionable whether it is possible to make a reliable production *e.g.* of thermoelectric modules based on this material.

## Supplementary Material

Further experimental data, tables and figures. DOI: 10.1107/S2052252516012707/lc5067sup1.pdf

## Figures and Tables

**Figure 8:**
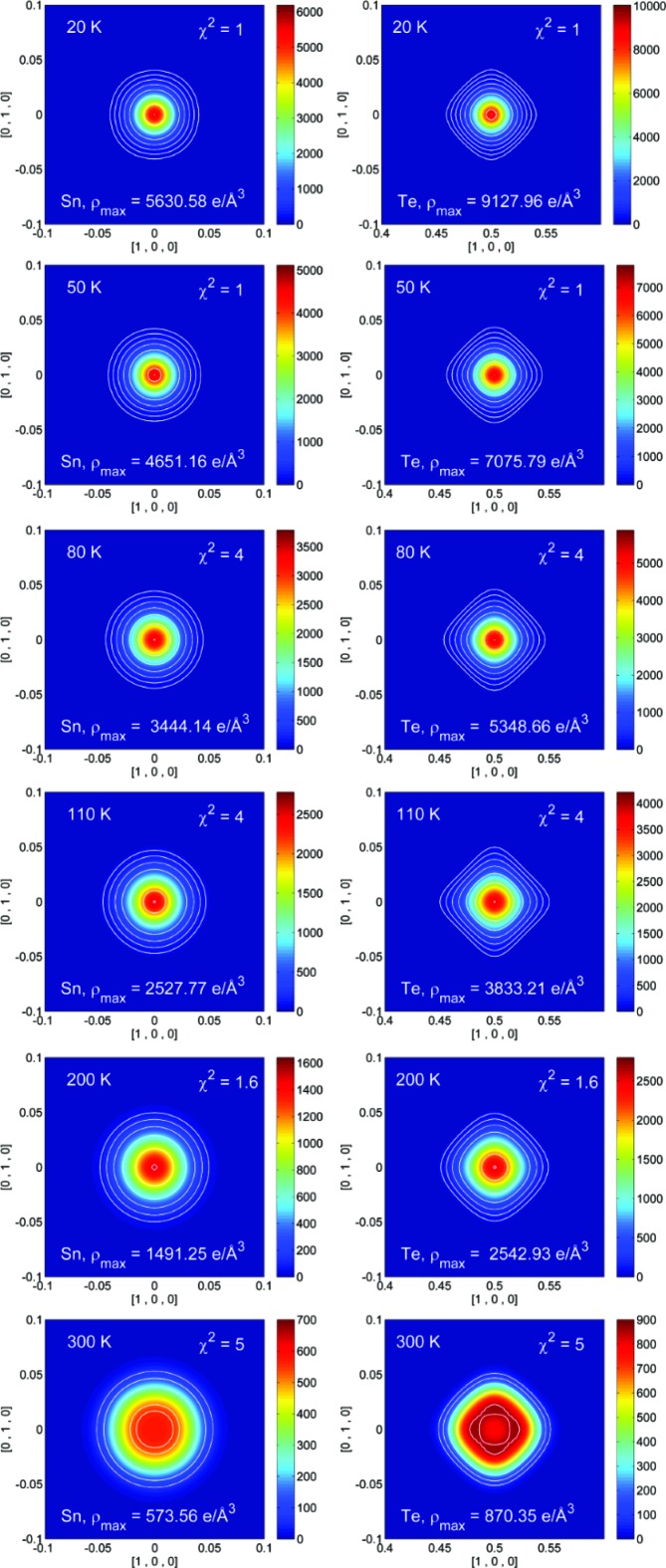
NXMEM electron-density maps for Sn (left) and Te (right) in the (001) plane from 20 to 300 K for sample *B*. Contour lines have been set at 64, 128, 256, 512, 1024, 2048, 4096 and 8192 e Å^−3^. An additional contour line has been added as guide to locate the maximum corresponding to the nuclear position.
