# Supplementary material for: Carrier concentration dependence of structural disorder in thermoelectric Sn1−xTe
Source: IUCrJ. 2016 Aug 22;3(Pt 5):377–88. doi: 10.1107/S2052252516012707 (PMC5391859; doi:10.1107/S2052252516012707)
Supplement: Supplementary file 1 [file m-03-00377-sup1.pdf]

Supporting information

S1. Sample characterization

S1.1. Hall coefficient and resistivity measurements

Hall and resistivity measurements on sample B have been performed with a PPMS (Physical Property Measurement System) from Quantum Design in the range 10 - 300 K. A bar of 8.442 mm x 3.596 mm x 0.63 mm was obtained from the ingot of sample B. The Au wires used for the resistivity measurement were placed 3.45 mm apart centered around the middle along the length of the sample. The Hall coefficient was calculated according to:

$$R_H = \frac{E_H}{jB} = \frac{V_H A}{IlB}$$

where E_H is the Hall field, B is the magnetic field, V_H is the Hall potential, $j = I/A$ is the current density i.e. the current divided by the sample cross section, and l is the separation of the transverse voltage leads. The carrier concentration at 77 K was estimated through:

$$p_{77K} = -\frac{1}{R_{H,77\mathrm{K}}e}$$

The same apparatus was used for electrical resistivity measurements.

S1.2. X-Ray diffraction

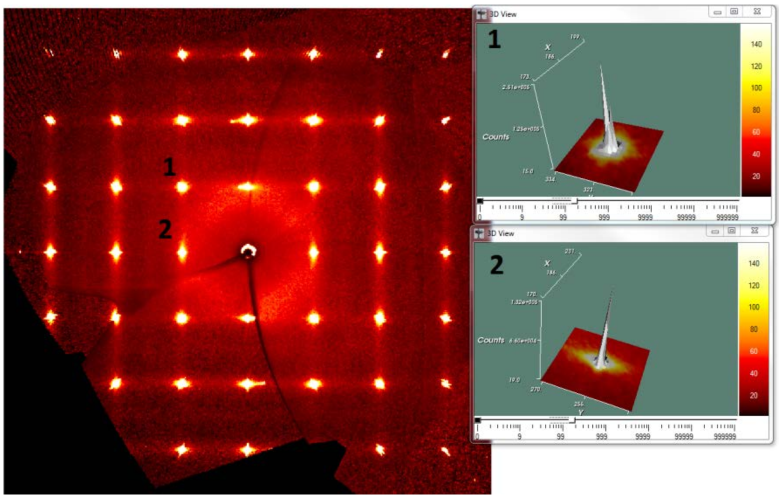

Figure S1 Reconstruction of the (0kl) plane, sample A, at 300 K with enlargement on the (022) reflection (1) and on the (002) reflection (2) using an in-house Bruker II diffractometer.

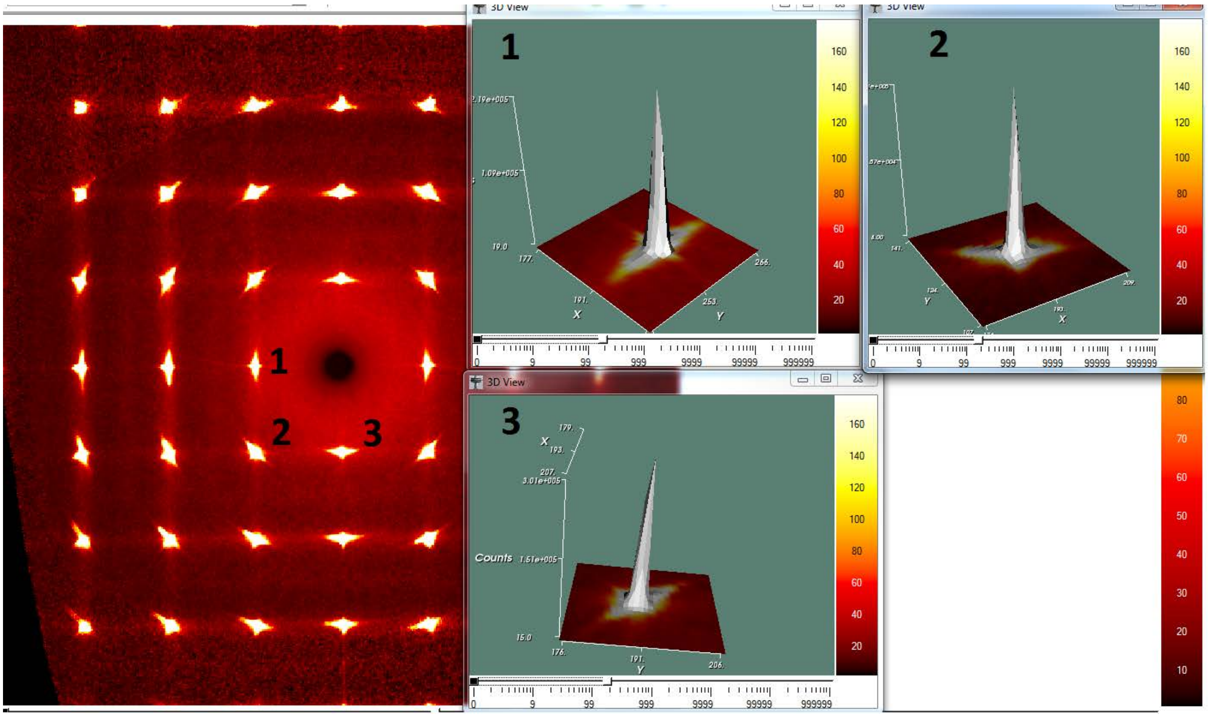

Figure S2 Reconstruction of the (0kl) plane, sample B, at 300 K with enlargement on the (020) reflection (1), on the (022) reflection (2) and on the (002) reflection (3) using an in-house Bruker II diffractometer.

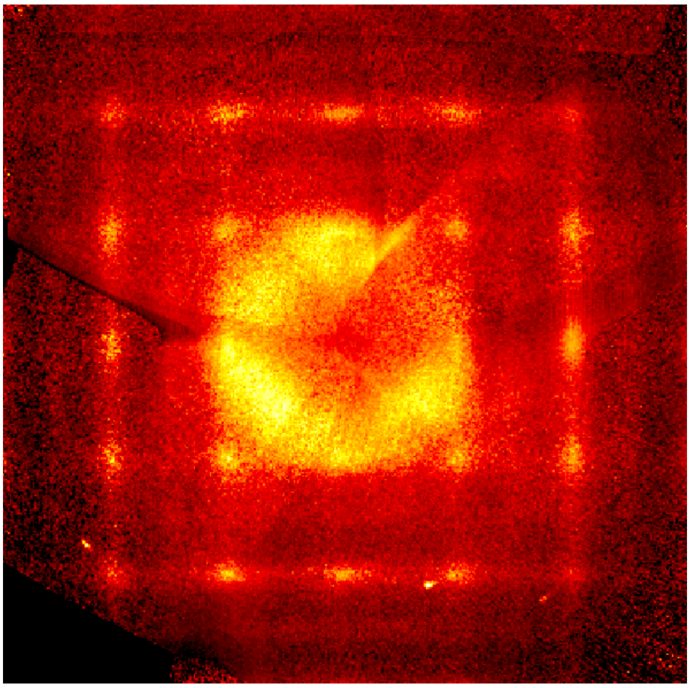

Figure S3 Reconstruction of the (0.5kl) plane, sample A, at 300 K using an in-house Bruker II diffractometer.

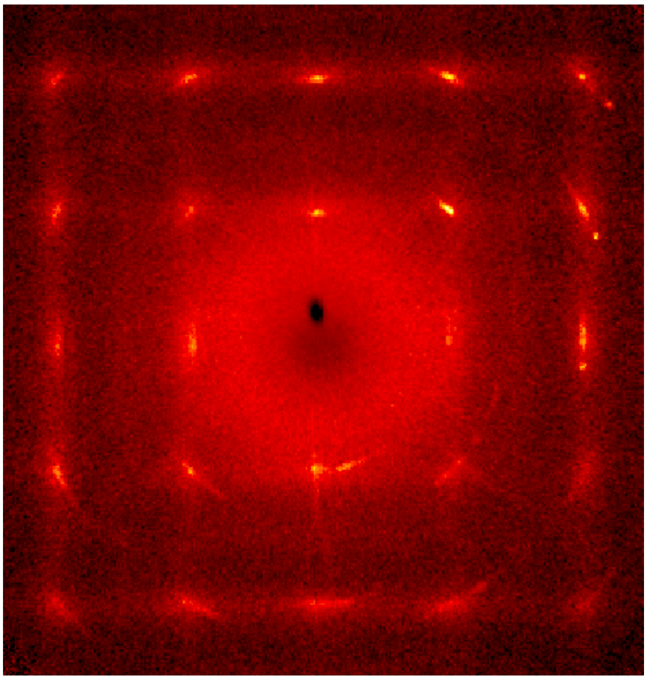

Figure S4 Reconstruction of the (0.5kl) plane, sample B, at 300 K using an in-house Bruker II diffractometer.

Single crystal X-ray data collections on sample A and sample B were carried out at BL02B1, SPring8 using ω-scans. For sample A frames were collected with Δω = 15° and one frame with Δω = 5° at T = 20 K, 200 K, 300 K, 400 K. Additionally 2 frames with Δω = 15° were collected at 50 K, 75 K, 110 K. For sample B 10 frames with Δω=15° and 3 frames with Δω = 5° were collected at 20 K, 50 K, 80 K, 110 K, 200 K, 300 K. The data have been merged in Sortav.

Refinements have been carried out in Jana2006 in which:

$$R_{obs} = \frac{\sum_i \left\| F_i(obs) \right| - \left| F_i(calc) \right\|}{\sum_i \left| F_i(obs) \right|} \quad ; \quad wR_{obs} = \sqrt{\frac{\sum_i w_i \left(F_i(obs)^2 - F_i(calc)^2 \right)^2}{\sum_i w_i F_i(obs)^4}} \quad ;$$

$$GOF = \sqrt{\frac{\sum_i w_i \left(\left| F_i(obs) \right| - \left| F_i(calc) \right| \right)^2}{n-p}}$$

Table S1 Experimental details (sample A, single crystal)

Crystal system				cubic			
Space group				$Fm\bar{3}m$			
Z				4			
λ (Å)				0.49912			
M (mm^{-1})				7.3			
Crystal size (equivalent radius) (μm)				40			
$(\sin\theta/\lambda)_{max}$ (Å^{-1})				1.2			
Temperature (K)	20	50	75	110	200	300	400
Empirical Formula	$Sn_{0.9779}Te$	$Sn_{0.977}Te$	$Sn_{0.9789}Te$	$Sn_{0.9798}Te$	$Sn_{0.9787}Te$	$Sn_{0.9795}Te$	$Sn_{0.9803}Te$
Formula weight	243.69	243.58	243.81	243.91	243.78	243.88	243.97

(g mol^{-1})							
F_{000}	403.58	403.40	403.78	403.96	403.74	403.9	404.06
a (Å)	6.2804(8)	6.282(2)	6.2871(17)	6.2907(18)	6.3017(10)	6.314(1)	6.3226(11)
V (Å^3)	247.72	247.91	248.51	248.94	250.25	251.66	252.75
ρ, g cm^{-3}	6.5340	6.5261	6.5163	6.5080	6.4705	6.4368	6.4115
$N_{measured}$	2006	344	520	347	2026	1982	2111
$N_{measured}$ (I>3σ)	2006	344	518	347	2026	1982	2057
N_{unique}	125	96	113	111	127	127	126
N_{unique} (I>3σ)	125	96	108	102	127	127	126
Average redundancy	16.0	3.6	4.6	3.1	16	15.6	16.8
Completeness %	100	76.8	90.4	88.8	100	100	99.2
R_1=R_{merge}	0.0311	0.0454	0.0576	0.0358	0.0342	0.0238	0.0274
R_{obs} (I>3σ)	0.0130	0.0318	0.0302	0.0291	0.0142	0.0153	0.0105
wR_{obs} (I>3σ)	0.0182	0.0410	0.0268	0.0236	0.0153	0.0151	0.0121
GOF (I>3σ)	1.32	1.69	1.00	0.89	1.07	1.09	0.78
Δρ (e Å^{-3}) (I>3σ)	-3.0 /1.3	-4.2 / 4.2	-4.5 / 3.3	-3.6 / 4.0	-1.2 / 1.2	-1.5 /0.9	-0.7 / 0.5
Δρ (e Å^{-3}) (I>3σ) weight scheme applied	-2.0 / 0.4	-3.7 / 2.9	-2.9 /2 .8	-2.4 / 2.0	-0.8 /0.4	-1.1 / 0.6	-0.4 / 0.2

Table S2 Experimental details (sample B, single crystal)

Crystal system	cubic					
Space group	$Fm\bar{3}m$					
Z	4					
λ (Å)	0.49972					
M (mm^{-1})	7.3					
Crystal size (equivalent radius) (μm)	25					
$(\sin\theta/\lambda)_{max}$ ($Å^{-1}$)	1.0					
Temperature (K)	20	50	80	110	200	300
Empirical Formula	$Sn_{0.973}Te$	$Sn_{0.978}Te$	$Sn_{0.9822}Te$	$Sn_{0.9828}Te$	$Sn_{0.9846}Te$	$Sn_{0.9858}Te$
Formula weight (g mol^{-1})	243.10	243.70	244.20	244.27	244.48	244.62
F_{000}	402.60	403.60	404.44	404.56	404.92	405.16
a (Å)	6.2892(16)	6.2912(18)	6.295(2)	6.300(2)	6.312(2)	6.327(2)
V ($Å^3$)	248.76	249.00	249.45	250.05	251.48	253.28
ρ, g cm^{-3}	6.6701	6.6714	6.6672	6.6717	6.6748	6.6849
$N_{measured}$	1195	1193	1194	1190	1208	1807
$N_{measured}$ (I>3σ)	1194	1190	1194	1187	1207	1796
N_{unique}	78	78	77	77	77	78
N_{unique} (I>3σ)	78	78	77	77	77	78
Average redundancy	15.3	15.3	15.5	15.4	15.7	23.1
Completeness %	100	100	98.7	98.7	98.7	98.7
R_1=R_{merge}	0.0426	0.0430	0.0423	0.0414	0.0394	0.0439
R_{obs} (I>3σ)	2.43	2.32	2.27	2.37	2.28	2.16
wR_{obs} (I>3σ)	3.69	3.30	3.09	3.33	2.51	2.67
GOF (I>3σ)	1.20	1.05	1.90	2.12	0.93	1.73
Δρ (e $Å^{-3}$) (I>3σ)	-2.1/4.1	-2.0/4.0	-2.0/3.5	-2.1/4.3	-1.9/2.7	-1.4/1.9

$\Delta\rho$ (e Å^{-3}) (I>3σ) weight scheme applied	-0.8/1.9	-0.8/1.7	-1.5/2.9	-1.6/2.6	-0.5/0.9	-1.1/1.3

Table S3 Extinction correction parameters as derived from Jana2006, harmonic model.

Sample A, 20 K	
Type 1, Lorentzian	G_{ISO} = 0.007109 ± 0.002369
Type 1, Gaussian	G_{ISO} = 0.006454 ± 0.002068
Type 2	RHO_{ISO} =0.040079 ± 0.013945

Sample B, 20 K	
Type 1, Lorentzian	G_{ISO} = 0.004580 ± 0.0066
Type 1, Gaussian	G_{ISO} = 0.004262 ± 0.0066011
Type 2	RHO_{ISO} =0.026832 ± 0.035168

Conventional PXRD data were collected from 300 to 800 K on a Rigaku Smartlab equipped with a copper source. The measurements were executed in a closed system under Argon.

S1.3. Differential thermal analysis (DTA)

DTA analysis has been performed with a Netsch STA 449 C Jupiter from 30 °C to 650 °C and back to 30 °C at a heating rate of 10 °C/min under Argon flux. The DTA curve shows a large broad partially irreversible exothermic transition. A small kink is visible at 233 °C which can be attributed to melting of metallic Sn which is present as a tiny impurity in the PXRD pattern.

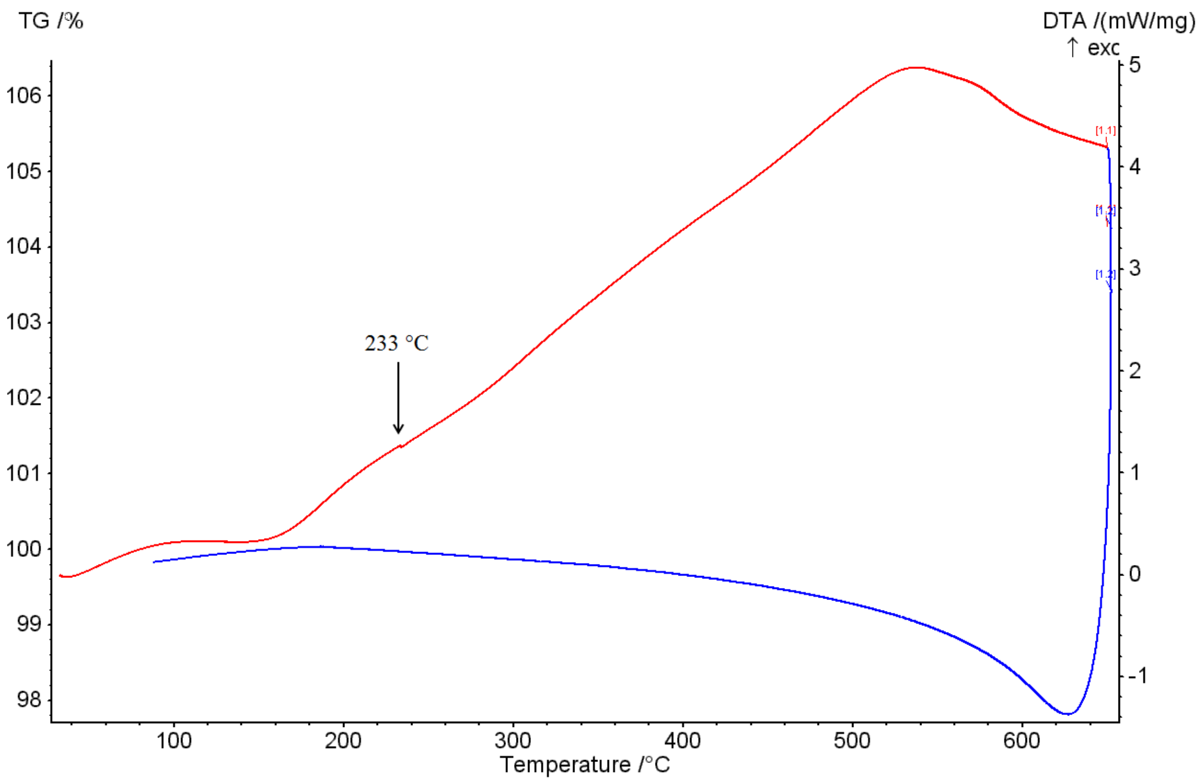

Figure S5 DTA curve from 30 °C to 650 °C (red curve) and from 650 °C to 30 °C (blue curve). A broad exothermic transition appears at around 150 °C and continues for all the range of temperature considered. The small endothermic kink at 233°C is due melting of the Sn impurity.

S2. Results and discussions

S2.1. Cell parameters

Table S4 Thermal expansion with temperature for sample A and B (single crystal data)

Sample A

T (K)	a (Å)
20	6.2804(8)
50	6.282(2)
75	6.2871(17)
110	6.2907(18)
200	6.3017(10)
300	6.3135(10)
400	6.3226(11)

Sample B (single crystal data from 20 to 300 K, conventional PXRD data from 320 to 800 K)

T (K)	a (Å)
20	6.2892(16)
50	6.2912(18)
80	6.295(2)
110	6.300(2)
200	6.312(2)
300	6.327(2)
320	6.32921(5)
340	6.33156(5)
360	6.33409(3)
380	6.33664(4)
400	6.33913(4)

420	6.34206(4)
440	6.34447(4)
460	6.34652(4)
480	6.34842(4)
500	6.34916(4)
520	6.34827(5)
540	6.34777(5)
560	6.34746(4)
580	6.34889(3)
600	6.35198(3)
620	6.35619(4)
640	6.35886(4)
660	6.36205(1)
680	6.36434(4)
700	6.36693(5)
720	6.37001(7)
740	6.37166(6)
760	6.37375(6)
780	6.37610(6)
800	6.37664(7)

S2.2. Harmonic model

Table S5 Isotropic ADPs and site occupancy factor of Sn from the harmonic model

Sample A

T (K)	U_{iso} Sn (Å^2)	U_{iso} Te (Å^2)	Occupancy(Sn)	R_1	R_{obs}	wR_{obs}
20	0.00482(7)	0.00292(6)	0.9779(8)	0.0310	0.0130	0.0182
50	0.00597(16)	0.00349(14)	0.977(2)	0.0454	0.0318	0.0410
75	0.00752(11)	0.00449(9)	0.9789(12)	0.0576	0.0302	0.0268
110	0.00963(10)	0.00589(8)	0.9798(12)	0.0355	0.0291	0.0236
200	0.01509(6)	0.00967(5)	0.9787(8)	0.0342	0.0142	0.0153
300	0.02184(6)	0.01448(5)	0.9795(8)	0.0238	0.0153	0.0151
400	0.02858(5)	0.01931(4)	0.9803(7)	0.0274	0.0105	0.0121

Sample B

T (K)	U_{iso} Sn (Å^2)	U_{iso} Te (Å^2)	Occupancy(Sn)	R_1	R_{obs}	wR_{obs}
20	0.0091(2)	0.0063(2)	0.973(2)	0.0426	0.0245	0.0370
50	0.0099(2)	0.00734(19)	0.978(2)	0.0430	0.0234	0.0331
80	0.0114(2)	0.00841(18)	0.9822(18)	0.0423	0.0227	0.0309
110	0.01272(19)	0.00915(16)	0.9828(12)	0.0414	0.0237	0.0333
200	0.01752(19)	0.01217(17)	0.9846(12)	0.0400	0.0228	0.0251
300	0.02442(18)	0.01712(16)	0.9858(16)	0.0439	0.0216	0.0267

S2.3. Anharmonic model

Table S6 Gram Charlier coefficients and site occupancy factor of Sn. Te is refined harmonically.

Sample A

T (K)	D_{1111} Sn (Å^4)	D_{1122} Sn (Å^4)	Occupancy(Sn)
20	0.00015(3)	0.000009(9)	0.980(9)
50	0.00024(9)	-0.00000(5)	0.980(3)
75	0.00014(6)	-0.000036(3)	0.9797(14)
110	0.00008(9)	-0.00000(4)	0.9802(15)
200	0.000010(5)	0.000046(19)	0.9801(10)
300	0.00005(6)	0.00003(3)	0.9799(10)
400	-0.00020(9)	0.00008(4)	0.9797(9)

Sample B

T (K)	D_{1111} Sn (Å^4)	D_{1122} Sn (Å^4)	Occupancy(Sn)
20	0.00072(16)	0.00042(7)	0.980(2)
50	0.00038(15)	0.00030(6)	0.984(2)
80	0.00014(12)	0.00023(5)	0.9860(19)
110	0.000067(15)	0.00024(6)	0.986(2)
200	0.00021(17)	0.00019(8)	0.9860(13)
300	0.00018(2)	0.00020(9)	0.9870(18)

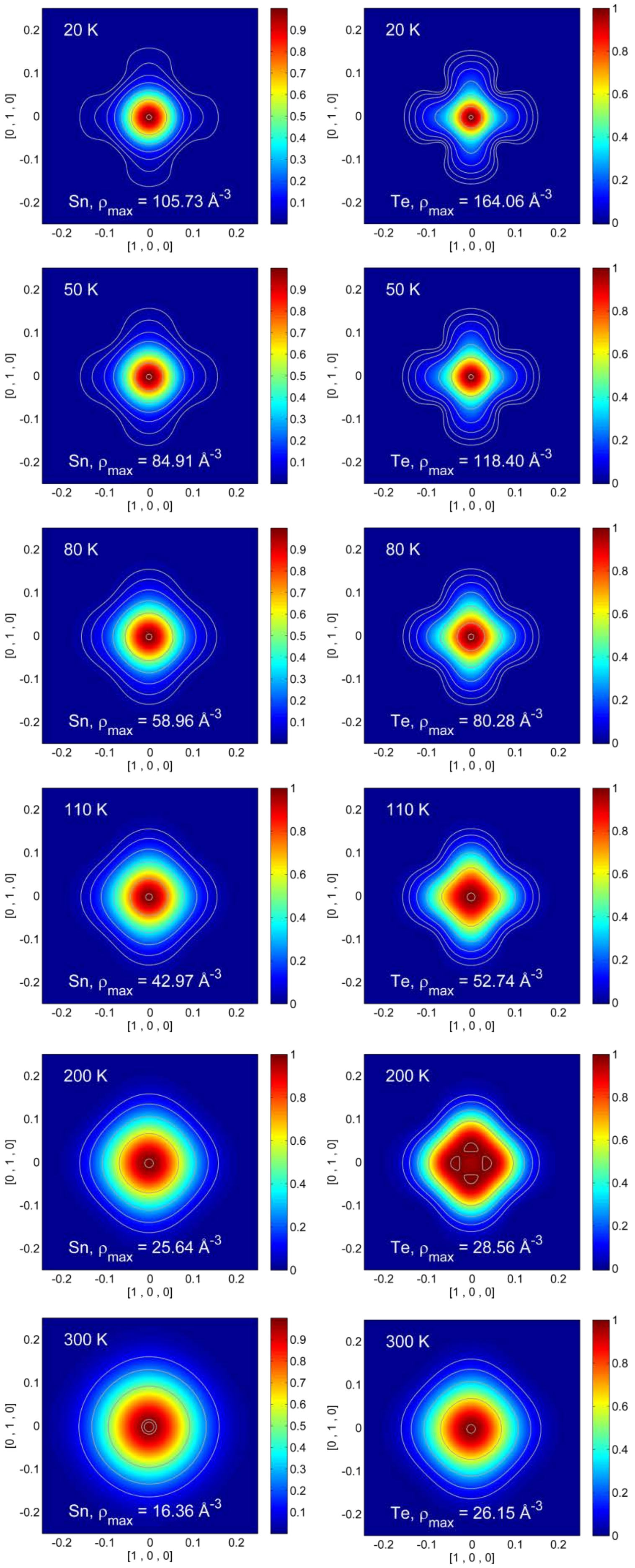
20 K
Sn, ρmax = 105.73 Å-3
20 K
Te, ρmax = 164.06 Å-3
50 K
Sn, ρmax = 84.91 Å-3
50 K
Te, ρmax = 118.40 Å-3
80 K
Sn, ρmax = 58.96 Å-3
80 K
Te, ρmax = 80.28 Å-3
110 K
Sn, ρmax = 42.97 Å-3
110 K
Te, ρmax = 52.74 Å-3
200 K
Sn, ρmax = 25.64 Å-3
200 K
Te, ρmax = 28.56 Å-3
300 K
Sn, ρmax = 16.36 Å-3
300 K
Te, ρmax = 26.15 Å-3
[1 , 0 , 0]
[0 , 1 , 0]

Figure S6 Nuclear probability density functions for sample B in the anharmonic model with both Sn and Te refined through Gram-Charlier coefficients. Both Sn and Te have been translated in the (0,0) position. The plane of the map is the (001). The values have been normalized to 1 and the contour are plot as $(2^n)/A$ where $1 \leq n \leq 8$ and A is the unnormalized value of maximum of nuclear density. An additional contour has been added at 0.99 in the normalized scale to show the nuclear position. Despite the large correlations it is indicative to notice evident non-spherical features, especially for the Te atom. At 200 K the Te nuclear maxima are not in the high symmetry position.

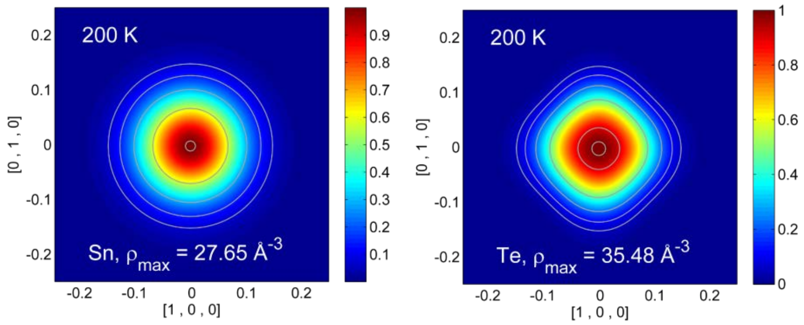

Figure S7 Nuclear probability density functions for sample B at 200 K in the anharmonic model with Te refined through Gram-Charlier coefficients while keeping Sn harmonic. The maps have been plotted with the same settings as Figure S2.

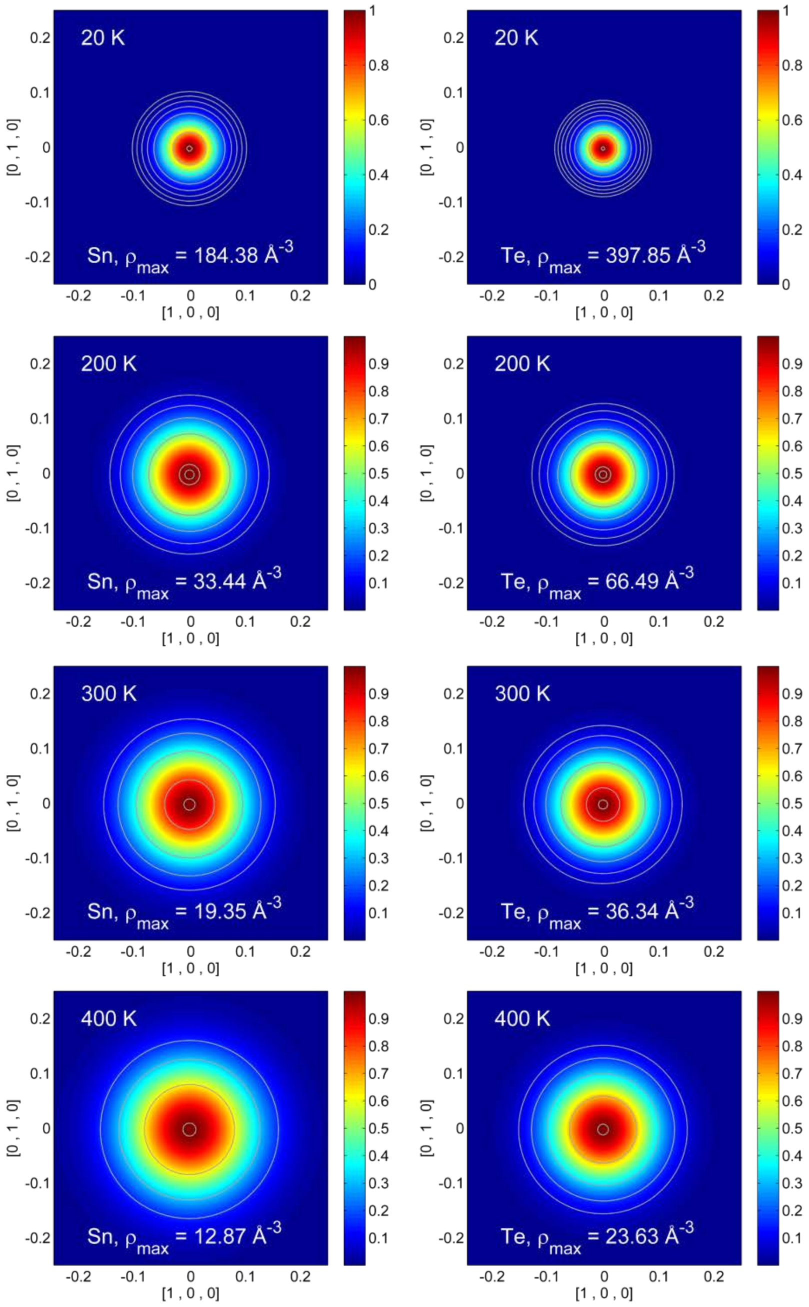

Figure S8 Nuclear probability density functions for sample A in the anharmonic model with both Sn and Te refined with Gram-Charlier coefficients. The maps have been plotted with the same settings as Figure S2. No deviations from sphericity are seen for Sn and Te.

S3.4 Maximum entropy method

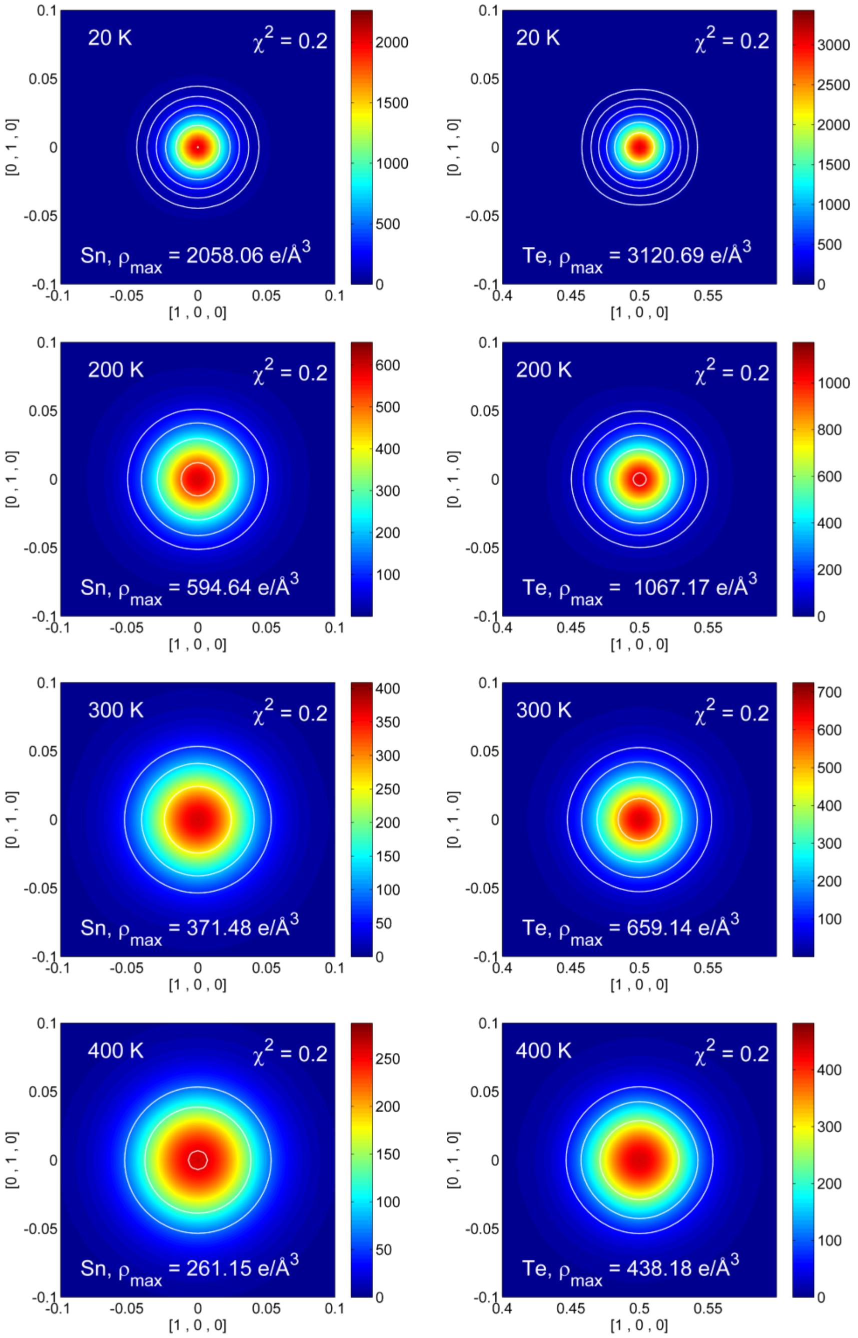

Figure S9 MEM electron density maps of Sn (left) and Te (right) in the (001) plane from 20 to 400 K for sample A. Contour values have been set to [64, 128, 256, 512, 1024, 2048] eÅ^{-3}.

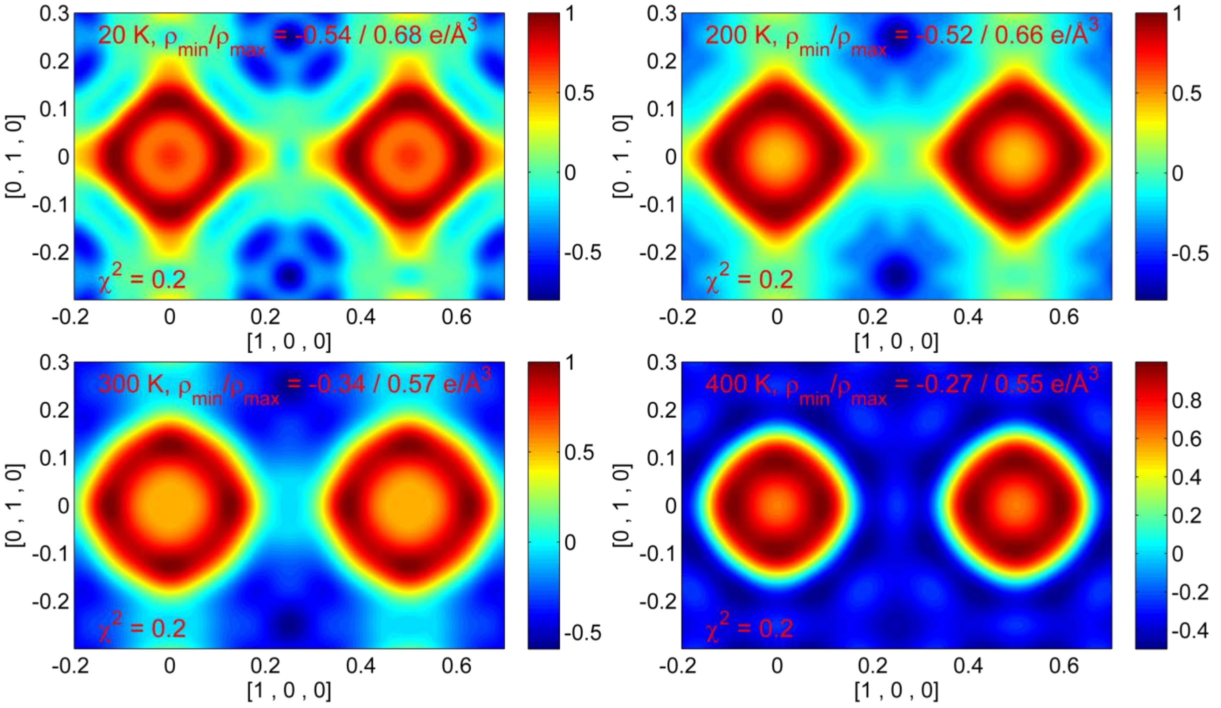

Figure S10 Fourier difference (F_{obs}-F_{MEM}) maps in the (100) plane at different temperatures for sample A. The values have been normalized between $\Delta\rho_{max}$ and $\Delta\rho_{min}$.

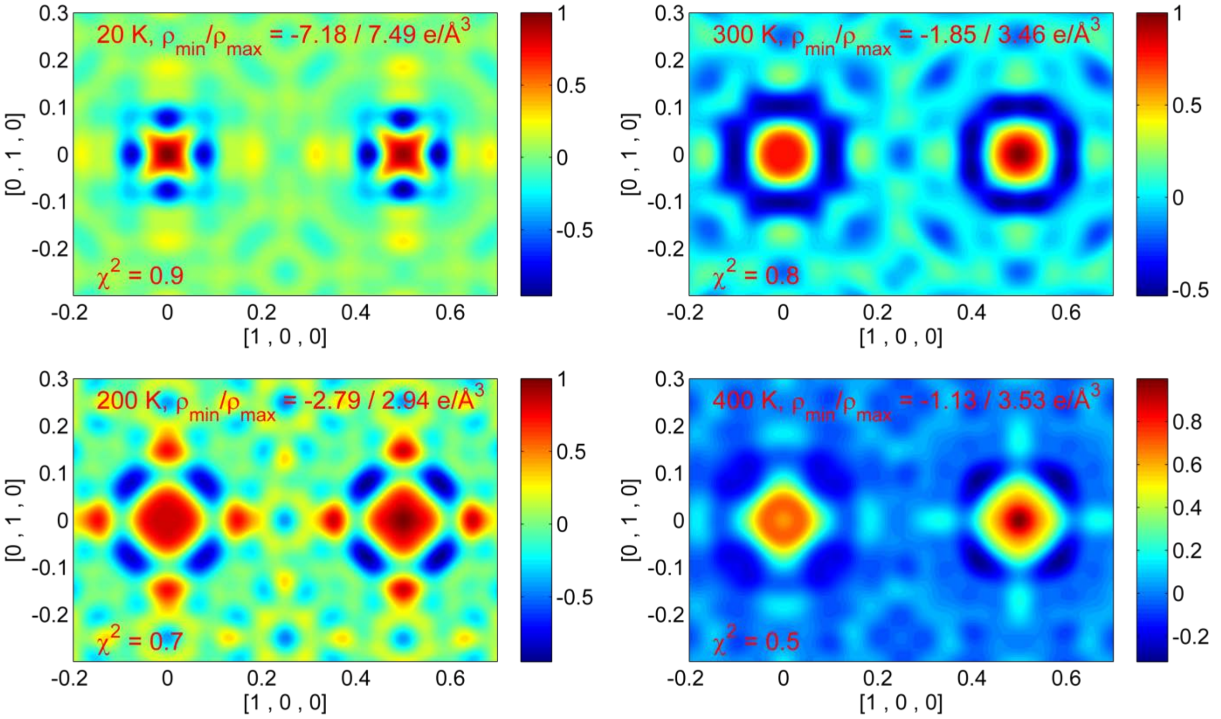

Figure S11 Fourier difference (F_{obs}-F_{NXMEM}) maps at different temperatures for sample A. The values have been normalized between $\Delta\rho_{max}$ and $\Delta\rho_{min}$

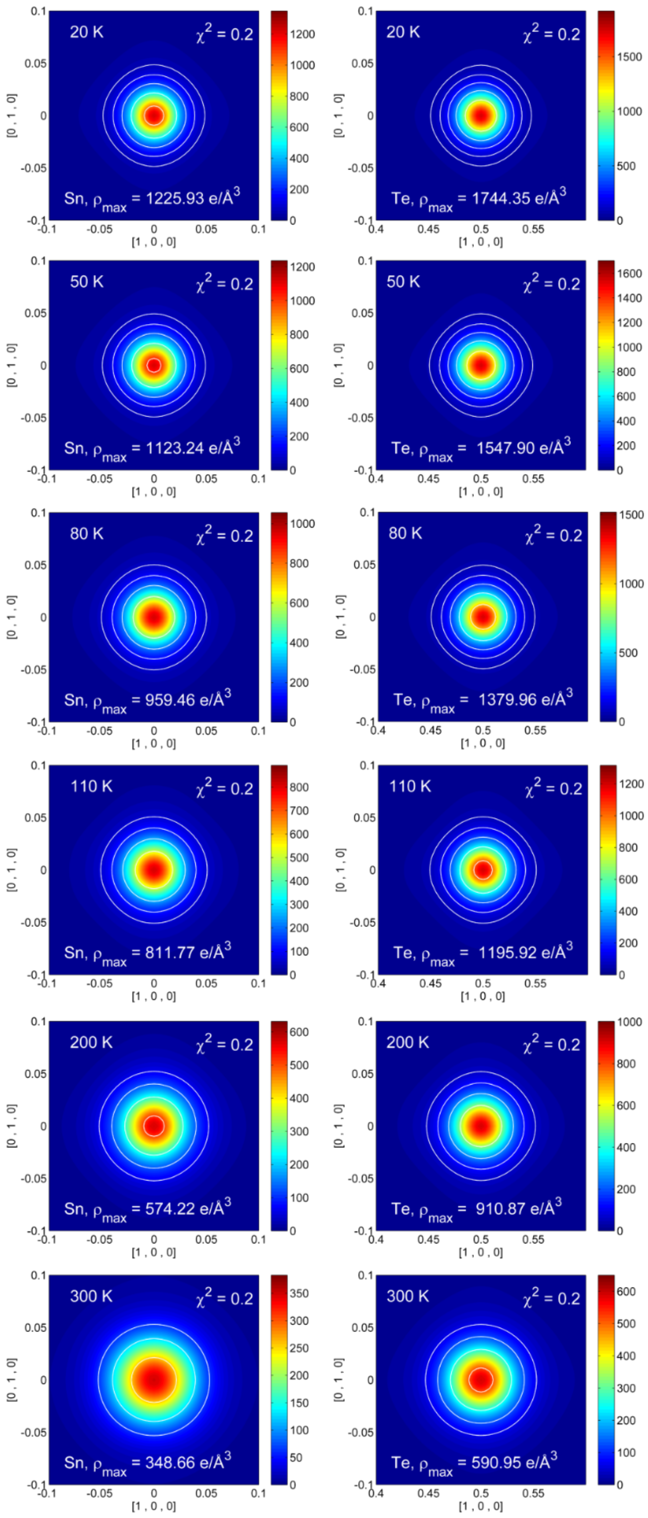

Figure S12 MEM electron density maps of Sn (left) and Te (right) in the (001) plane from 20 to 300 K for sample B. Contour values have been set to [64, 128, 256, 512, 1024, 2048] eÅ^{-3}.

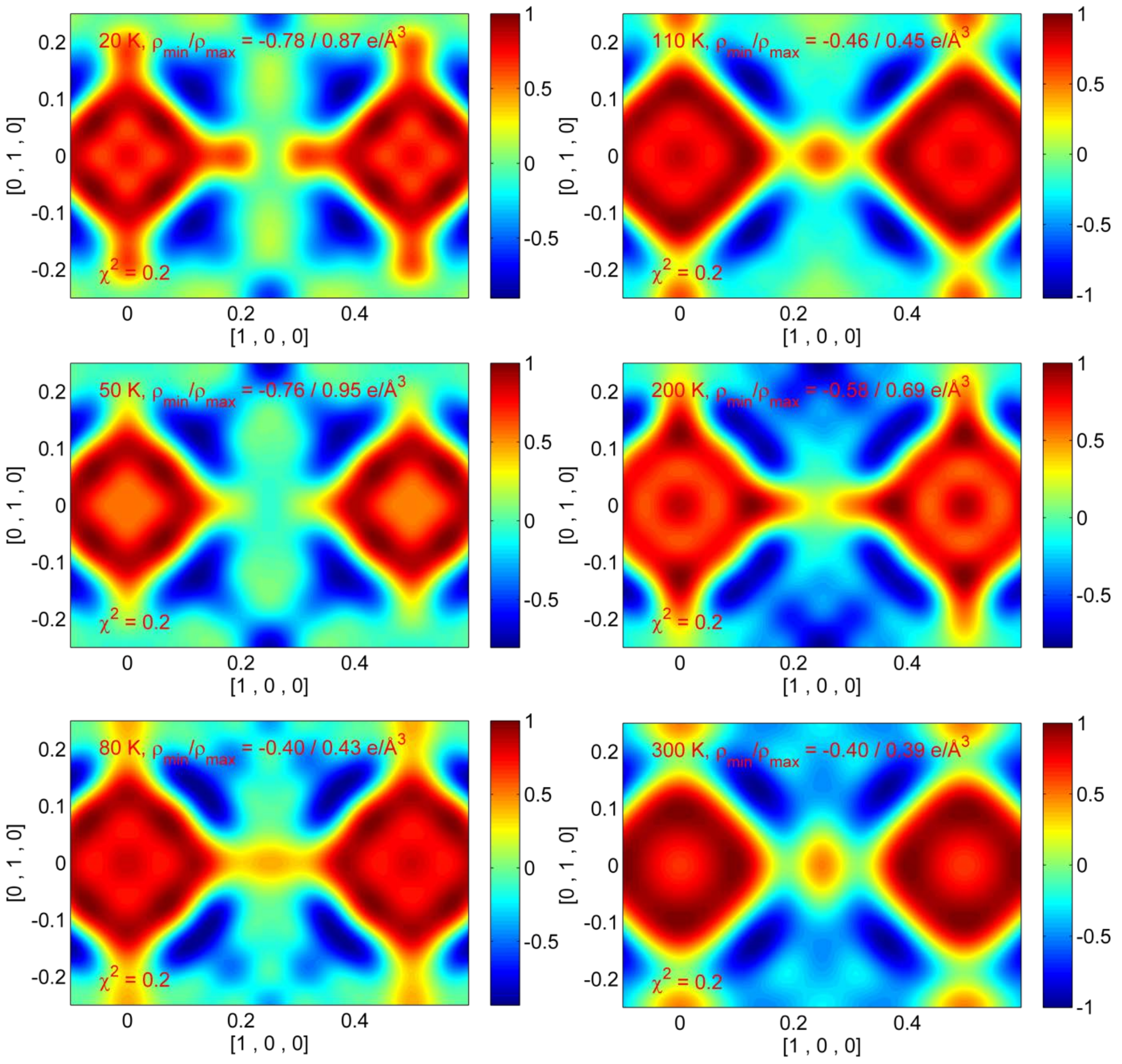

Figure S13 Fourier difference (F_{obs}-F_{MEM}) maps at different temperatures for sample B. The values have been normalized between $\Delta\rho_{max}$ and $\Delta\rho_{min}$.

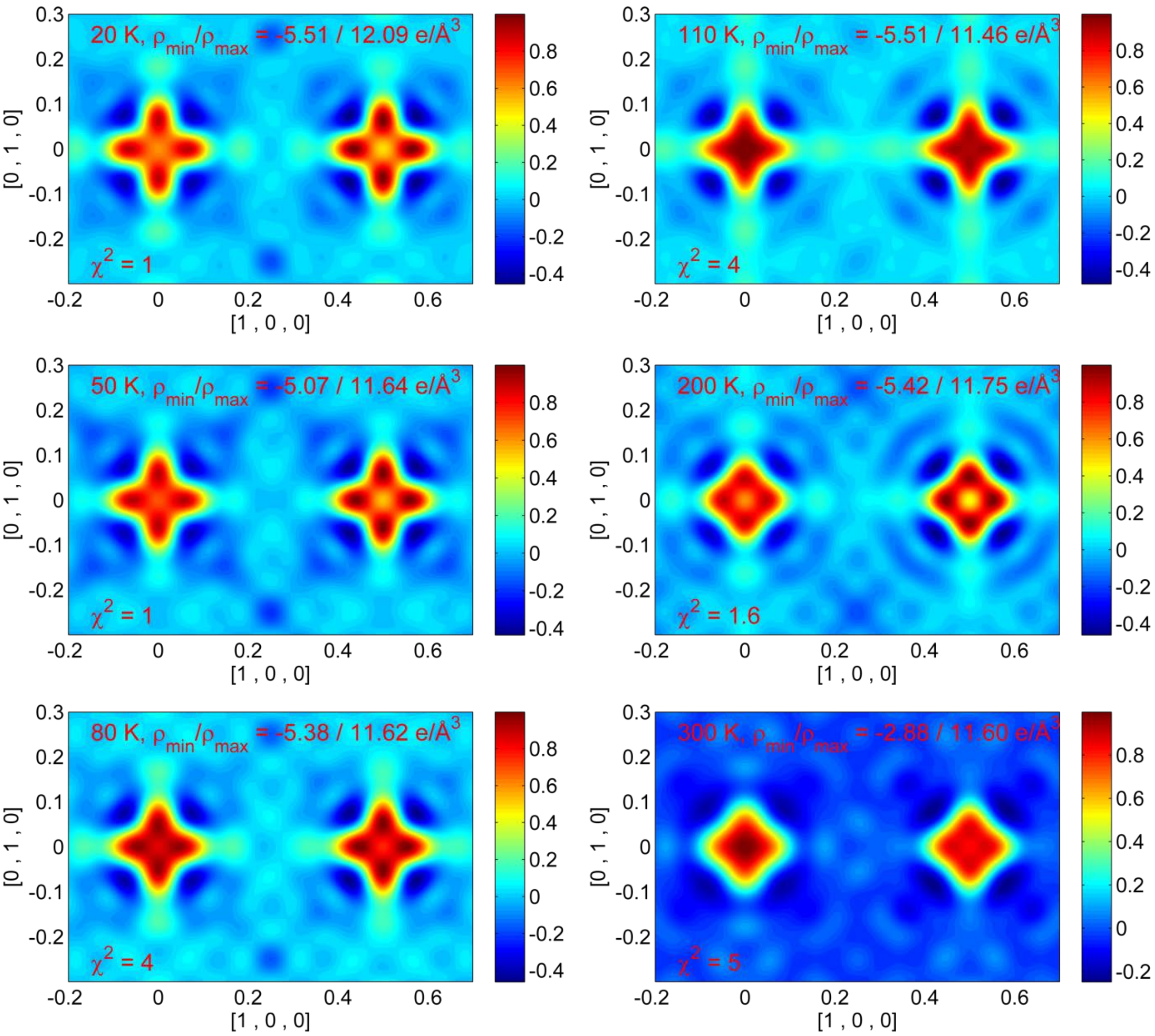

Figure S14 Fourier difference (F_{obs}-F_{NXMEM}) maps at different temperatures for sample B. The values have been normalized between $\Delta\rho_{max}$ and $\Delta\rho_{min}$.

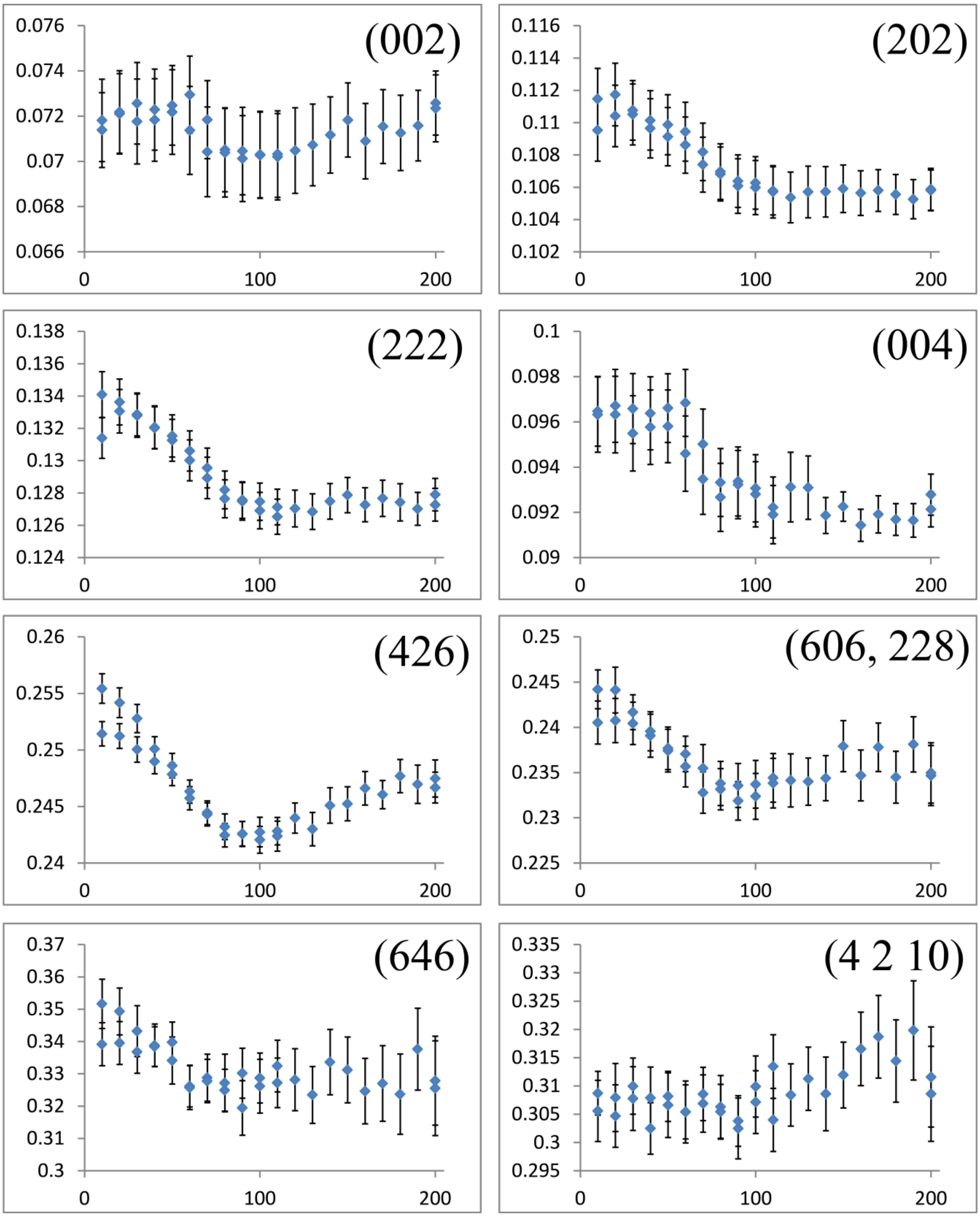

Figure S15 Integral breadth β (deg), (y axis) as function of Temperature (K). The low order reflections show a slight broadening approaching 10 K. (002) and (004) should not split or broaden if a phase transition $Fm\bar{3}m \rightarrow R3m$ occurs. At high 2θ values the broadening appears negligible.

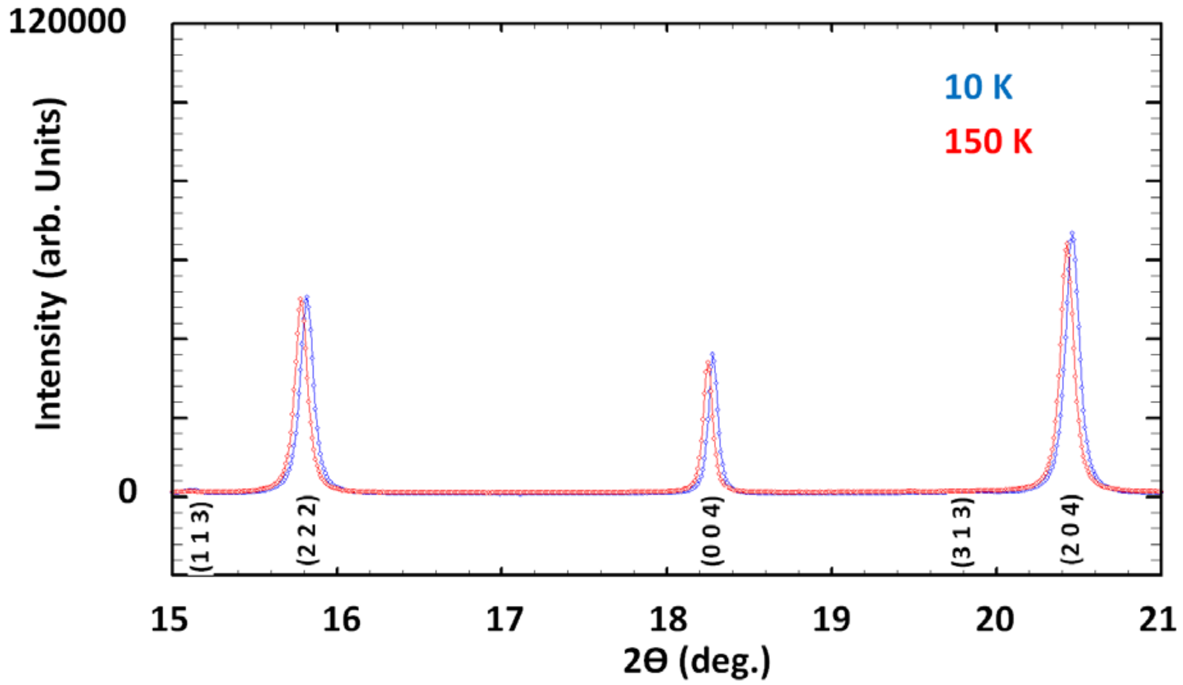

Figure S16 Comparison of low order reflections at 10 and 150 K.

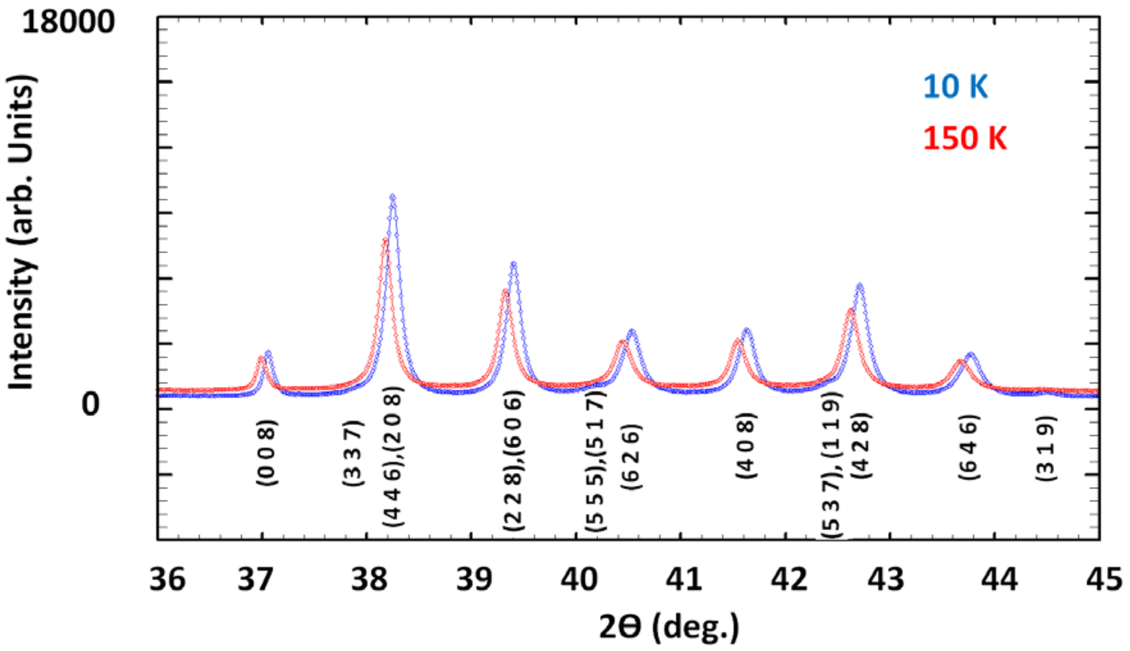

Figure S17 Comparison of high order reflections at 10 and 150 K.
